# Effect of Lactoferrin Supplementation on Inflammation, Immune Function, and Prevention of Respiratory Tract Infections in Humans: A Systematic Review and Meta-analysis

**DOI:** 10.1093/advances/nmac047

**Published:** 2022-04-27

**Authors:** Bronwyn S Berthon, Lily M Williams, Evan J Williams, Lisa G Wood

**Affiliations:** Priority Research Centre for Healthy Lungs, Hunter Medical Research Institute, The University of Newcastle, Newcastle, Australia; Priority Research Centre for Healthy Lungs, Hunter Medical Research Institute, The University of Newcastle, Newcastle, Australia; Priority Research Centre for Healthy Lungs, Hunter Medical Research Institute, The University of Newcastle, Newcastle, Australia; Priority Research Centre for Healthy Lungs, Hunter Medical Research Institute, The University of Newcastle, Newcastle, Australia

**Keywords:** lactoferrin, humans, inflammation, immune function, respiratory tract infection

## Abstract

Lactoferrin (Lf) is a glycoprotein present in human and bovine milk with antimicrobial and immune-modulating properties. This review aimed to examine the evidence for the effect of Lf supplementation on inflammation, immune function, and respiratory tract infections (RTIs) in humans. Online databases were searched up to December 2020 to identify relevant, English-language articles that examined the effect of Lf supplementation in human subjects of all ages, on either inflammation, immune cell populations or activity, or the incidence, duration, or severity of respiratory illness or RTIs. Twenty-five studies (*n* = 20 studies in adults) were included, of which 8 of 13 studies (61%) in adults reported a decrease in at least 1 systemic inflammatory biomarker. Immune function improved in 6 of 8 studies (75%) in adults, with changes in immune cell populations in 2 of 6 studies (33%), and changes in immune cell activity in 2 of 5 studies (40%). RTI outcomes were reduced in 6 of 10 studies (60%) (*n* = 5 in adults, *n* = 5 in children), with decreased incidence in 3 of 9 studies (33%), and either decreased frequency (2/4, 50%) or duration (3/6, 50%) in 50% of studies. In adults, Lf reduced IL-6 [mean difference (MD): –24.9 pg/mL; 95% CI: –41.64, –8.08 pg/mL], but not C-reactive protein (CRP) [standardized mean difference: –0.09; 95% CI: –0.82, 0.65], or NK cell cytotoxicity [MD: 4.84%; 95% CI: –3.93, 13.60%]. RTI incidence was reduced in infants and children (OR: 0.78; 95% CI: 0.61, 0.98) but not in adults (OR: 1.00; 95% CI: 0.76, 1.32). Clinical studies on Lf supplementation are limited, although findings show 200 mg Lf/d reduces systemic inflammation, while formulas containing 35–833 mg Lf/d may reduce RTI incidence in infants and children, suggesting improved immune function. Future research is required to determine optimal supplementation strategies and populations most likely to benefit from Lf supplementation. This trial was registered at PROSPERO (https://www.crd.york.ac.uk/prospero/display_record.php?ID=CRD42021232186) as CRD42021232186.

## Teaser

**Statement of Significance**: Experimental trials highlight roles for lactoferrin supplementation in reducing inflammation and modulating immune function. This is the first systematic literature review to summarize the available evidence for the effects of lactoferrin supplements on inflammation, immune function, and respiratory tract infections in both adults and children.

## Introduction

Lactoferrin (Lf) is a nonheme iron-binding glycoprotein from the transferrin family that is present in exocrine biological fluids such as breast milk, tears, bronchial secretions, and gastrointestinal fluids and is an important component of human and bovine milk (1). Lf is released from activated neutrophil granules; thus, concentrations in plasma and feces increase during infection and inflammation due to neutrophil recruitment (2). Human Lf (hLf) is analogous in terms of structure and function to bovine Lf (bLf) and, while concentrations of Lf are significantly higher in human milk than bovine milk, bLf can be efficiently extracted from bovine milk in large quantities. bLf is increasingly being used as a nutritional supplement in a range of patients and conditions, for which it is generally regarded as safe and well tolerated (3).

Lf plays an important role in host defense through a variety of physiological functions, including antiviral, antimicrobial, antioxidant, and immunomodulatory activities (4). These protective functions of Lf can be either dependent or independent of its ability to bind iron (2). Recent evidence suggests a role for Lf in protecting against common viral infections, by enhancing type I IFN production, NK cell activity, and type 1 T-helper (Th1) cell cytokine responses (5). Several in vitro studies have reported Lf treatment inhibits virus growth, cellular entry, and cytopathic effect following inoculation with common respiratory viruses, including respiratory syncytial virus (6, 7) and influenza virus (8, 9). In addition, colostrum, which contains high concentrations of Lf (∼7 g/L) (10) has been shown to be protective against respiratory tract infections (RTIs) in adults and children (11, 12). Recently, preliminary evidence using pseudo-viruses suggests that Lf may be protective against emerging viruses such as severe acute respiratory syndrome coronavirus 2 (SARS-CoV-2), the virus responsible for causing the pandemic coronavirus disease 2019 (COVID-19) (13, 14).

A limited number of systematic literature reviews have previously assessed the safety and efficacy of Lf supplementation in preventing confirmed viral infections (15), neonatal care (16–18), dermatology (19), *Helicobacter pylori* infection (20), and in pregnant women with iron deficiency anemia (21). However, to our knowledge, there have been no systematic reviews of the literature to date that investigated the effects of Lf supplementation on systemic inflammation, immune function, and/or RTIs. Therefore, the aim of this systematic review was to identify all relevant publications, synthesize the current knowledge, and evaluate the effects of Lf supplementation on inflammation, immune function, and RTIs in humans. To this end, this systematic review aimed to determine whether Lf supplementation affects systemic or airway inflammatory biomarkers, peripheral immune cell population numbers, immune cell activity or function, and the incidence, duration, or severity of RTIs. Secondary aims were to establish what dose of Lf is required for beneficial effects on either inflammation, immune function, or protection against RTIs.

## Methods

This systematic review followed the Preferred Reporting Items for Systematic Reviews and Meta-Analyses (PRISMA) guidelines. The protocol for this systematic review was prospectively registered with PROSPERO (National Institute for Health Research, University of York, UK; CRD42021232186; https://www.crd.york.ac.uk/prospero/display_record.php?ID=CRD42021232186).

### Search strategy

Relevant articles were identified and retrieved via online database searches using medical subject headings (MeSH) and keywords related to Lf supplementation interventions, inflammation, immune function, and RTIs, limited to the English language and human subjects. Online databases included MEDLINE, EMBASE (Excerpta Medica Database), and Cumulative Index to Nursing and Allied Health Literature (CINAHL) (each from inception up to 15 December 2020). (See **Supplemental Table 1** for the MEDLINE search strategy.) To ensure literature saturation, reference lists of included studies and relevant reviews identified through the initial search were hand-searched for additional relevant articles. Key articles retrieved via online databases and hand-searching reference lists were used for further searches using the Web of Science database Cited Reference Function. The results of Cited Reference searches were narrowed using the keywords lactoferrin, inflammation, immune function, and infection.

### Population, Intervention, Comparison, Outcomes, and Study (PICOS) criteria

#### Populations

Studies were included if they investigated Lf supplementation in adults or children, including subjects who were overweight or obese, and populations with specific diseases or medical conditions. There were no restrictions on age, sex, or ethnicity. Studies were excluded if they were performed in participants in a critical care setting, including subjects with chronic infections such as hepatitis or HIV/AIDS, or in subjects with cancer or sepsis.

#### Interventions

Included studies examined the effects of Lf supplementation in humans at any dosage or duration. Interventions delivering a Lf supplement in combination with other active ingredients were included only if the dose of Lf was specified. Lf interventions were classified according to the type, recombinant human Lf (rhLf), or bLf. There was no specified intervention or follow-up length and no additional restrictions by type of study setting. Interventions where Lf was delivered via routes other than oral or intranasal administration, such as vaginally or topically, or where the intervention dose of Lf was not specified were excluded. Experimental ex vivo studies that did not involve direct Lf supplementation in humans were also excluded.

#### Comparators

Studies with nonsupplemented control groups, including a placebo or other supplement type, or no control group were included.

#### Outcomes

Lf intervention studies were included if their outcomes included markers of systemic or airway inflammation such as fraction of exhaled NO (FeNO), interleukins (IL-6, IL-8), C-reactive protein (CRP), and TNF-α; markers of immune function such as immune cell population percentage [including innate lymphoid cells (ILC1, ILC2, ILC3 with and without natural cytotoxicity receptor (NCR+/–)], granulocytes (eosinophils, neutrophils), dendritic cells [blood dendritic cell antigen (BDCA) 1 (BDCA-1), BDCA-3, plasmacytoid], NK cells, and lymphocytes [B cells, cluster of differentiation (CD) (CD4+) T cells, activated CD4+ T cells, CD8+ T cells, activated CD4+ T cells, Treg cells, TCR-B T cells, γδ-T cells]; immune cell activity (NK cell activity); markers of inflammation and immune function in human cell ex vivo studies following Lf supplementation in vivo; incidence of either lower, upper, or unspecified RTIs (e.g., incidence of cold or flu-like symptoms, incidence of bronchitis or bronchiolitis, detection of respiratory viruses, etc.); or symptom severity scores/duration of RTIs. Studies were excluded if outcomes were solely immune changes within the gastrointestinal tract (GIT),

#### Studies

The study designs included were randomized controlled trials (RCTs), including cluster RCTs and randomized controlled crossover trials (RCXTs), nonrandomized controlled trials (NRCTs) including nonrandomized controlled crossover trials (NRCXTs), and noncontrolled trials (NCTs). Animal studies, observational studies, case studies, conference abstracts, study protocols, and literature reviews were excluded.

#### Screening process

Following the search strategy, retrieved studies were evaluated for relevancy using Covidence systematic review software (Veritas Health Innovation, Melbourne, Australia; www.covidence.org). The number of records acquired from each database were noted, with duplicate studies noted and removed. Two independent reviewers (BSB, LMW) used inclusion and exclusion criteria to assess retrieved studies based on title and abstract. Irrelevant articles were removed, and full texts of the remaining articles were retrieved and assessed for relevancy by 2 independent reviewers (BSB, LMW). If there was any disagreement between the 2 reviewers regarding the relevancy of an article, a third independent reviewer decided outcome (EJW).

#### Data extraction

A customized data extraction template table was used to collect relevant data from each article, including author, publication year, country, population, baseline characteristics (age, sex, and sample size), type of intervention (rhLf or bLf, Lf dose, placebo composition, and intervention duration), outcomes of interest [including markers of systemic or airway inflammation markers of immune function (means and SDs) before and after the supplementation period for each group], incidence frequency, duration and severity of RTIs, conflicts of interest, limitations, and conclusions. Authors were contacted to retrieve unpublished data; when data could not be obtained, WebPlotDigitizer (version 4.4; https://apps.automeris.io/wpd/) was used to extract data from published figures. When SD values for any outcomes of interest were not reported, they were calculated from the reported SEs or 95% CIs, and when medians were reported, sample means and SDs were estimated using the method proposed by Wan et al. (22). For data synthesis and reporting of RTIs, data collected from each article were categorized into standard outcomes, including incidence, episode frequency, episode duration, cumulative duration, and episode severity. Incidence refers to the number of subjects affected. Episode frequency refers to the number of discrete illness events per subject during the study period. Episode duration refers to the average duration (days) of all reported episodes, where cumulative duration refers to the total number of days of illness during the study period. Episode severity refers to the average severity of reported episodes. Data extraction tables are available on request by contacting the corresponding author via e-mail.

#### Risk of bias

Retrieved studies were assessed for quality using the Academy of Nutrition and Dietetics Quality Criteria Checklist, a standardized critical appraisal tool designed by the American Dietetic Association, by 1 reviewer (BSB). This tool assessed study reliability, validity, and generalizability. Studies receiving negative quality scores (response to ≥6 validity questions was “no”) were excluded from the review. The evidence level for each article was defined according to the study design based on the National Health and Medical Research Council (NHMRC) of Australia levels of evidence hierarchy (23).

### Meta-analysis

Review Manager (RevMan, version 5.3; The Nordic Cochrane Centre, The Cochrane Collaboration, 2014) software was used to perform meta-analysis. Data synthesis was performed according to the statistical guidelines provided in the Cochrane Handbook for Systematic Reviews of Interventions) (24). Random-effects model meta-analyses were performed using inverse variance to calculate mean difference (MD), standardized MD (SMD), or OR effect size and corresponding 95% CIs. Heterogeneity was assessed using chi-square test (*P* < 0.1 considered to indicate significant heterogeneity) and *I*^2^ parameter (30–60% indicating moderate, 50–90% indicating substantial, and 75–100% indicating considerable heterogeneity). Subset analysis was performed when considerable heterogeneity was identified, in groups of studies according to their outcome variables (change from baseline or postintervention) or by the population (sex, pregnancy status, age). Pooling of study data for meta-analysis across subsets/subgroups was performed for outcomes where limited included studies reported the outcome. The Cochrane Handbook for Systematic Reviews of Interventions was used to determine whether it was appropriate to include data from crossover studies in the meta-analyses, based on the ability to rule out significant carryover effects (24). Funnel plots were visually examined for asymmetry to identify publication bias.

## Results

### Study selection

**FIGURE 1:**
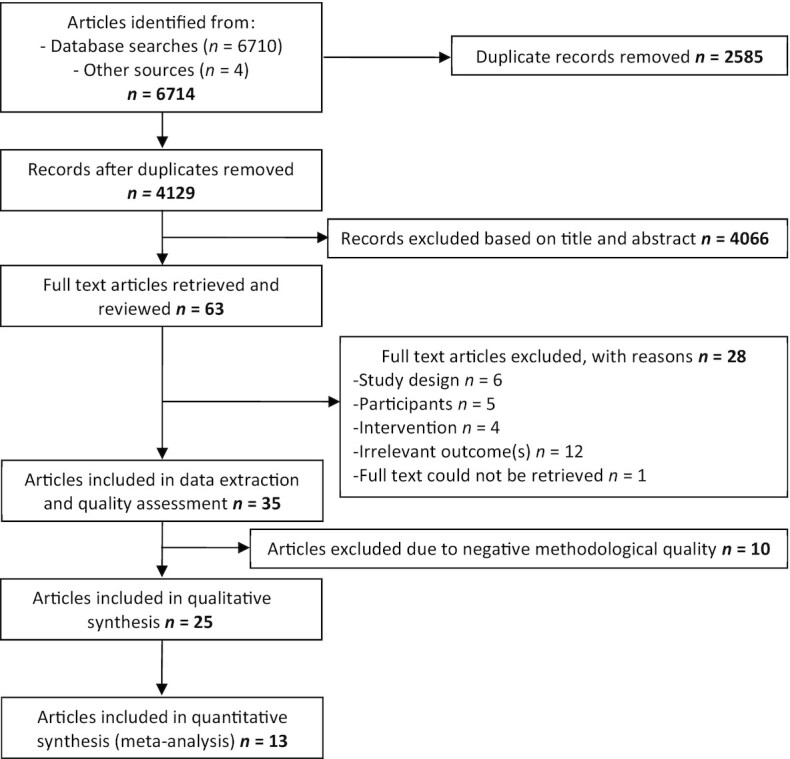
PRISMA flow chart of systematic review on the effect of lactoferrin supplementation on inflammation, immune function, and prevention of respiratory tract infections. PRISMA, Preferred Reporting Items for Systematic Reviews and Meta-Analyses.

Following the search strategy and selection process (**Figure 1**), 63 studies were retrieved for full-text screening, of which 28 were excluded. Data extraction was performed on the remaining 35 articles, which were then assessed for methodological quality (**Supplemental Table 2**). Ten articles deemed to have negative methodological quality according to the quality criteria checklist were excluded (25–34), leaving 25 articles eligible for inclusion in the review and qualitative analysis. The most common contributing factors to negative quality ratings were as follows: intervention not described in adequate detail or subject compliance with the intervention not measured (Question (Q) 6, *n* = 9, 90%), lack of identification and discussion of study biases and limitations (Q9, *n* = 9, 90%), insufficient detail on subject inclusion/exclusion criteria or baseline health and demographic characteristics (Q2, *n* = 8, 80%), lack of blinding (Q5, *n* = 8, 80%), statistical analysis inappropriate or inadequately described (Q5, *n* = 8, 80%), and likely bias due to funding sources/affiliations or apparent conflict of interest (Q10, *n* = 7, 70%). The characteristics of excluded studies (*n* = 10 studies) and the effects of Lf supplementation on review outcomes are presented in **Supplemental Table 3**.

### Description of included studies

Of the 25 included studies, 19 were RCTs (76%) including 1 RCXT, 3 were NRCTs (12%) including 1 NRXT, and 3 were NCTs (12%). Included trials were published from 2007 to 2020 (median: 2014). Most trials were performed in Asia (*n* = 10, 40%), followed by Europe (*n* = 8, 32%), Australia (*n* = 5, 20%), and the United States (*n* = 2, 8%). The number of subjects in each trial ranged from 8 to 451 (median: 61), with a total of 2329 subjects included in this review. Most trials were performed in adults (≥18 y) (*n* = 20, 80%, 1447 subjects) with 3 trials (12%, 716 subjects) in infants and 2 trials (8%, 166 subjects) in children. All trials in infants and children were performed in healthy subjects. The subject population of most trials in adults was healthy subjects (*n* = 9, 36%), followed by pregnant and nonpregnant females with hereditary thrombophilia (HT) (*n* = 2, 8%) or iron deficiency (ID) (*n* = 2, 8%), with 1 trial each in type 2 diabetes (T2D), polycystic ovarian syndrome (PCOS), bedridden neurological patients, atopic dermatitis (AD), patients with colonic polyps, periodontal disease, and atopic keratoconjunctivitis (AK). All trials used bLf in doses ranging from 32.4 mg/d to 3 g/d (median: 225 mg/d), delivered either alone [total, *n* = 14, 56%; tablet, *n* = 6 (35–40); capsule, *n* = 7 (41–47); powder, *n* = 1 (48)], or in combination with other ingredients [enteral/infant feeding formula, *n* = 6 (49–54); whey protein, *n* = 2 (55, 56); synbiotic, *n* = 2 (57, 58); and myo-inositol, *n* = 1 (59)]. The comparators/controls included inert placebo formulations (*n* = 5), matched control feeding formula (*n* = 5), calcium phosphate (*n* = 4), a different dose of bLf (*n* = 3), routine care (ferrous sulfate, *n* = 2), synbiotic (*n* = 1), and prebiotic (*n* = 1), while 3 trials were noncontrolled. The intervention duration ranged from 1 to 60 wk (median: 12 wk). The effects of bLf supplementation on systemic inflammatory biomarker outcomes were measured in 13 trials (52%) (35, 40, 41, 43, 45–48, 53–55, 57, 59) (**Table 1**), 8 trials (32%) (36–39, 42, 44, 48, 53) measured immune function outcomes (**Table 2**), and 10 trials (40%) (39, 42, 49–54, 56, 58) reported on RTI outcomes (**Table 3**). Airway inflammation was not measured as an outcome in any included trials. In terms of methodological quality, 6 trials were rated as positive (24%) and 19 were rated as neutral quality (76%) (Supplemental Table 2).

**TABLE 1:** Summary of included trials examining the effect of lactoferrin on systemic inflammatory biomarkers in adults and children^1^

First author, year (country) (ref)	Participants, age, *n*	Intervention, daily dose	Protocol	Control, daily dose	Duration	Effect of intervention on inflammation
Adults						
Bharadwaj, 2010 (USA) (41)	Healthy females, 45-60 y, *n* = 31	R-ELF bLf capsule, 250 mg/d	2 × 125 mg bLf capsule once daily, NFD	Calcium tablet, 100% RDA, NFD	180 d	↔ CRP↓ TNF-α^2^↓ IFN-γ^2^↓ IL-1β^2^↓ IL-6^2^↑ IL-12+p40^2^↑ IL-10^2^↔ TGF-β
Derosa, 2020 (Italy) (55)	T2D, >18 y, *n* = 117	WPI powder NFD with 0.7% bLf, 32.4 mg/d	1× sachet dissolved in water once daily, before breakfast	Placebo powder (caseins, 5 g/d) (per intervention protocol)	3 mo	↓ CRP^2,3^↓ TNF-α^2,3^↓ IL-6^2,3^
Fujishima, 2020 (Japan) (35)	AK, adults, 30.0 y (mean treatment), 28.1 y (mean control), *n* = 20	bLf EC tablet, 400 mg/d	2 × 100 mg bLf tablets twice daily, after breakfast and before bedtime	EC placebo tablets (lactose, NFD) (per intervention protocol)	12 wk	↔ ECP↔ IgE
Genazzani, 2014 (Italy) (59)	Healthy-weight PCOS females, 25.3 ± 2.2 y (mean ± SD), *n* = 24	Myoinositol powder 3 g/d, with bLf 200 mg/d and bromelain 40 mg/d, 200 mg/d	1× dose of powder (100 mg bLf) dissolved in water, twice daily at 10 am and 4 pm	N/A	12 wk	↓ CRP^3^
Lepanto, 2018 (Italy) (43)	Hereditary thrombophilia pregnant (6–8 wk of gestation) females, 34 ± 3 y (mean ± SD), *n* = 65	bLf capsule, 200 mg/d	1 × 100 mg bLf capsule twice daily, before meals	Ferrous sulfate (329.6 mg) 1× tablet daily, with a meal	Until delivery	↓ IL-6^3^
	Hereditary thrombophilia nonpregnant females, 31 ± 3 y (mean ± SD), *n* = 73	bLf capsule, 200 mg/d	1 × 100 mg bLf capsule twice daily, before meals	Ferrous sulfate (329.6 mg) 1× tablet daily, with a meal	30 d	↓ IL-6^3^
Paesano, 2010 (Italy) (46)	ID or IDA nonpregnant women, 19–45 y, *n* = 120	bLf capsule, 200 mg/d	1 × 100 mg bLf capsule twice daily, before meals	Ferrous sulfate, NFD, once daily, 520 mg/d	90 d	↔ IL-6
	ID or IDA pregnant women, 20–40 y, *n* = 60	bLf capsule, 200 mg/d	1 × 100 mg bLf capsule twice daily, before meals	Ferrous sulfate, NFD, once daily, 520 mg/d	30 d	↓ IL-6^3^
Paesano, 2012 (Italy) (45)	ID or IDA pregnant women, 20–40 y, *n* = 161	bLf capsule, 200 mg/d	1 × 100 mg bLf capsule twice daily, before meals	N/A	≥4 wk until delivery	↓ IL-6^3^
Rosa, 2020 (Italy) (47)	Hereditary thrombophilia pregnant (6–8 wk of gestation) females, 32 ± 3 y (mean ± SD), *n* = 31	bLf capsule, 200 mg/d	1 × 100 mg bLf capsule twice daily, before meals	bLf capsule, 200 mg/d, 1 × 100 mg twice daily, during meals	30 d	↓ IL-6^4^
Takeuchi, 2012 (Japan) (53)	Tube-fed bedridden neurological patients, 50–95 y, *n* = 61	Immune-enhancing enteral formula with bLf 1 g/L, ∼1 g/d	As per pre-study enteral feeding schedule, administered by tube (gastrostomy/nasogastric/esophagostomy)	Isocaloric regular enteral formula (per intervention protocol)	12 wk	↔ CRP
Tong, 2017 (Australia) (40)	AD, 32 ± 11 y (treatment, mean ± SD), 35 ± 10 y (control, mean ± SD), *n* = 35	bLf tablet, 250 mg/d	1 × 250 mg bLf tablet, once daily	Calcium phosphate (100 mg), 1× tablet daily	56 d	↔ CRP↔ IgE
Van Splunter, 2018(The Netherlands) (48)	Healthy females, 60–85 y, *n* = 30	bLf powder 1 g/d withGOS 2.64 g/d andcholecalciferol capsule 20 μg/d (day 0-21, bLf 1 g/d only; day 22-42, bLf 1 g/d + GOS 2.64 g/d; day 43-63, bLf 1 g/d + GOS 2.64 g/d + vitamin D 20 μg/d)	1× scoop of powder (1 g bLF) dissolved in water, once daily, after evening meal	Placebo powder (MDX, weight matched to intervention protocol) and capsule (MDX 250 mg/d)	63 d	↔ CRP↔ TNF-α↔ IL-1β↔ IL-6↔ IL-10↔ sVCAM↔ sICAM↔ IL-1Rα
West, 2012 (Australia) (57)	Male athletes, 33.9 ± 6.5 y (mean ± SD), *n* = 22	Synbiotic capsules with bLf 150 mg/d, and *Lactobacillus paracasei*, 1.38 × 10^9^ CFU/d, *B. animalis* 1.8 × 10^9^ CFU/d, *L. acidophilus* 1.38 × 10^9^ CFU/d, *L. rhamnosus* 1.38 × 10^9^ CFU/d, raftiline 270 mg/d, raftilose 30 mg/d, immunoglobulin 600 mg/d	3 × 50 mg bLf capsules daily, morning or evening with or without food	Prebiotic capsule (116 mg acacia gum) × 3 daily (per intervention protocol)	21 d	↔ IL-16↔ IL-18
Children						
Yen, 2011 (Taiwan) (54)	Healthy children, 2–6 y, *n* = 65	Growing-up formula with bLf 35 mg/100 mL, 70–85 mg/d	200–240 mL of formula, once daily, morning	Growing-up formula, 200–240 mL, once daily, morning	15 mo	↔ IFN-γ↔ IL-10

1 AD, atopic dermatitis; AK, atopic keratoconjunctivitis; bLf, bovine lactoferrin; CFU, colony forming units; CRP, C-reactive protein; EC enteric-coated; ECP, eosinophil cationic protein; GOS, galacto-oligosaccharide; ID, iron deficient; IDA, iron deficiency anemia; IL-1R, IL-1 receptor; MDX, maltodextrin; N/A, not applicable; NFD, not further described; NR, not reported; PCOS, polycystic ovarian syndrome; ref, reference; R-ELF, ribonuclease-enriched lactoferrin; sICAM, soluble intercellular adhesion molecule; sVCAM, soluble vascular cell adhesion molecule; T2D, type 2 diabetes; TGF, transforming growth factor; WPI, whey protein isolate; ∼, average intake; ↓, significant decrease; ↑, significant increase; ↔, no change.

2 Significant difference between groups in change compared with baseline.

3 Significant change within intervention group compared with baseline values.

4 Decreased IL-6 with bLf taken before meals; no change in IL-6 with bLf taken during meals.

**TABLE 2:** Summary of included trials examining the effect of lactoferrin on immune function in adults^1^

Author, year (country) (ref)	Participants, age, *n*	Intervention, daily dose	Protocol	Control, daily dose	Duration	Effect of intervention on immune function
Dix, 2018 (Australia) (42)	Healthy males, 18–65 y, *n* = 12	Microencapsulated bLf capsule, 200 mg/d or 600 mg/d	1 × 200 mg bLf or 3 × 200 mg bLf capsule/s, once daily, after breakfast	bLf capsules, 200 mg or 600mg/d (per intervention protocol)	4 wk/arm, 2-wk w/o	↓ %CD69+ on CD4+ cells^2^↔ %CD69+ on CD8+ cells↔ %CD4+ cells↔ %CD8+ cells
Ishikado, 2010 (Japan) (36)	Periodontal disease, 37–59 y, *n* = 14	Liposomal bLf tablet, 180 mg/d	4 × 45 mg tablets daily, NFD	N/A	4 wk	↔ White blood cell countLPS-stimulated PBMC release:↓ TNF-α^3^↓ IL-1β^3^↓ IL-6^3^
Kawakami, 2015 (Japan) (37)	Healthy adults, >65 y, *n* = 59	EC bLf tablet, 300 mg/d	3 × 100 mg bLF tablets once daily, after dinner	Placebo tablet (EC dextrin) 3× 100 mg tablets once daily, after dinner	3 mo	↔ %CD3+ cells↔ %CD4+ cells↔ %CD8+ cells↑ %CD16+ cells^3,4^↑ %CD56+ cells^3,4^↔ %CD80+ cells↑ %CD86+ cells^3,4^↔ CD4+/CD8+↔ Neutrophil phagocytic capacity (%)↑ NK cell activity^3,4^
Kozu, 2009 (Japan) (38)	Patients with colonic polyps, 40–75 y, *n* = 71	bLf tablet, 1.5 g/d or 3 g/d	6 × 250 mg bLf or 6 × 500 mg bLf tablets daily, NFD	Placebo tablet (D-sorbitol, maltitol, and corn starch) 6 × 1.5 g tablets daily	12 mo	↔ CD4+ cells↔ CD8+ cells↔ CD16+ cells↔ CD56+ cells↑ NK cell activity (%) (1.5 g/d)^4^
Mulder, 2008 (Australia) (44)	Healthy males, 31–52 y, *n* = 8	bLf capsule, 100 mg/d or 200 mg/d	1 × 100 mg bLf or 1 × 200 mg bLf capsule daily, with breakfast (day 0–6: placebo; day 7–13: 100 mg/d; day 14–20: 200 mg/d)	Calcium phosphate (200 mg) 1× capsule, once daily (days 0–6)	7 d/arm, no w/o	↑%CD69+ on CD3+ cells^3,5^↑%CD69+ on CD4+ cells^3,5^↑%CD69+ on CD8+ cells^3,5^
						↔ CD3+ cells↔ %CD3+ cells↔ CD4+ cells↔ %CD4+ cells↔ CD8+ cells↔ %CD8+ cells↔ B cells↔ NK cells↔ NK cell activity (%)
Oda, 2020 (Japan) (39)	Healthy adults with baseline NK cell activity ≈ 50% cytotoxicity, 20–65 y, *n* = 110	bLf tablet, 200 mg/d or 600 mg/d	6 × 33.3 mg bLf or 6 × 100 mg bLf tablets, with water, at bedtime	Placebo tablet (dextrin 250 mg) (per intervention protocol)	12 wk	↔ Neutrophil phagocytic capacity (%)↔ NK cell activity (%)
Takeuchi, 2012 (Japan) (53)	Tube-fed bedridden neurological patients, 50–95 y, *n* = 61	Immune-enhancing enteral formula with bLf 1 g/L, ∼1 g/d	As per pre-study enteral feeding schedule, administered by tube (gastrostomy/nasogastric/esophagostomy)	Isocaloric regular enteral formula (per intervention protocol)	12 wk	↔ Neutrophil phagocytic capacity (%)↓ Neutrophil sterilizing activity (%)^3^↔ NK cell activity (%)
Van Splunter, 2018 (The Netherlands) (48)	Healthy females, 60–85 y, *n* = 30	bLf powder 1 g/d, with GOS 2.64 g/d, and cholecalciferol capsule 20 μg/d (day 0–21, bLf 1 g/d only; day 22–42, bLf 1 g/d + GOS 2.64 g/d; day 43–63, bLf 1 g/d + GOS 2.64 g/d + vitamin D 20 μg/d)	1× scoop of powder (1 g bLF) dissolved in water, once daily, after evening meal	Placebo powder (MDX, weight matched to intervention protocol) and capsule (MDX 250 mg/d) (per intervention protocol)	63 d	↔ %pDC↔ %mDC↔ TLR2^6^↔ TLR4^6^↔ TLR7^6^↔ TLR9^6^TLR7/8 stimulated pDCs:↑ IL-6+ pDC^4,7^↔ IFN-α+ pDC↑ TNF-α+ pDC^3,8^

1 bLf, bovine lactoferrin; CD, cluster of differentiation; EC, enteric-coated; GOS, galacto-oligosaccharide; mDC, myeloid dendritic cell; MDX, maltodextrin; N/A, not applicable; NFD, not further described; PBMC; peripheral blood mononuclear cell; pDC, plasmacytoid dendritic cell; ref, reference; TLR, Toll-like receptor; w/o, washout; ∼, average intake; ↓, significant decrease, ↑, significant increase, ↔, no change.

2 Data from both treatments and dosages combined.

3 Significant change within intervention group compared with baseline values.

4 Significant difference between groups in change compared with baseline.

5 Significant difference between bLf 200 mg treatment and placebo in postintervention measurements.

6 Expression in isolated pDC and mDC cell populations.

7 Significant difference at day 21 following bLF only treatment.

8 Significant difference at day 42 following bLF and GOS treatment.

**TABLE 3:** Summary of included trials examining the effect of lactoferrin on respiratory tract infections/illness in adults and children^1^

Author, year (country) (ref)	Participants, age, *n*	Intervention, daily dose	Protocol	Control, daily dose	Duration	Effect of intervention on respiratory tract infections/illness
Adults						
Dix, 2018 (Australia) (42)	Healthy males, 18–65 y, *n* = 12	Micro-encapsulated bLf capsule, 200 mg/d or 600 mg/d	1 × 200 mg bLf or 3 × 200 mg bLf capsule/s once daily, after breakfast	bLf capsules, 200 mg/d or 600 mg/d (per intervention protocol)	4 wk/arm,2-wk w/o	↔ Viral infections (NFD)
Oda, 2020 (Japan) (39)	Healthy adults, 20–65 y, *n* = 187	bLf tablet, 200 mg/d or 600mg/d	6 × 33.3 mg bLf or 6 × 100 mg bLf tablets with water, at bedtime	Placebo tablet (dextrin 250mg) (per intervention protocol)	12 wk	Summer colds^2^↔ Incidence↓ Episode duration^3,4^↔ Episode frequency
Pregliasco, 2008 (Italy) (58)	Healthy adults, 36.9 ± 16.7 y (treatment, mean ± SD), 38.5 ± 19.2 y (control, mean ± SD), *n* = 144	Synbiotic powder sachet (*Lactobacillus plantarum* 10 × 10^9^ CFU, *L. rhamnosus* 10 × 10^9^ CFU, *B. lactis* 10 × 10^9^ CFU; FOS 3.0 g, with bLf 300 mg/d	1× sachet (300 mg bLf), dissolved in water once daily	Synbiotic powder sachet (*L. plantarum* 10 × 10^9^ CFU, *L. rhamnosus* 10 × 10^9^ CFU, *B. lactis* 10 × 10^9^ CFU; FOS 3.0 g (per intervention protocol)	90 d	↔ Incidence of total or individual respiratory illness (URTI, colds, influenza-like illness)↔ Episode frequency↔ Episode duration↔ Episode severity
Takeuchi, 2012 (Japan) (53)	Tube-fed bedridden neurological patients, 50–95 y, *n* = 61	Immune-enhancing enteral formula with bLf 1 g/L, ∼1 g/d	As per pre-study enteral feeding schedule, administered by tube (gastrostomy/nasogastric/esophagostomy)	Isocaloric regular enteral formula (per intervention protocol)	12 wk	↔ Incidence of RTIs (pneumonia, bronchitis, colds, URTIs)
Vitetta, 2013 (Australia) (56)	Healthy adults with ≥3 cold events in 6 mo, ≥18 y, *n* = 105	bLf capsule 200 mg with 100 mg Ig-rich fraction, 400 mg/d	2 × 200 mg bLf capsules daily, NFD	Calcium phosphate (300 mg) capsule, 2× daily	90 d	Colds^5^↓ Episode frequency^4^↔ Cumulative duration↔ Episode severity
Children						
Chen, 2016 (China) (49)	Healthy, weaned infants, 4–6 mo, *n* = 213	Infant formula with iron 4 mg/100 g and bLf 38 mg/100 g, ∼35.8 ± 3.7 mg/d	NR	Infant formula with iron 4 mg/100 g, NFD	3 mo	↓ Incidence respiratory illness:^4,6^↓ Runny nose, cough, and wheezing^4^↔ Nasal congestionEpisode duration:↓ Respiratory illness^4,6^↓ Runny nose^4^↔ Wheezing or cough
King, 2007 (USA) (50)	Healthy formula-fed infants, 0–4 wk, *n* = 52	Infant formula with iron 3 mg/L and bLf 850 mg/L, ∼833 mg/d	NR	Infant formula with iron 3 mg/L and bLf 102 mg/L, ∼100 mg/d, NR	12 mo	↔ URTI^7^ incidence↔ URTI^7^ cumulative duration↓ LRTI^8^ incidence^4^↔ LRTI^8^ cumulative duration
Li, 2019 (China) (51)	Healthy formula-fed infants, 10–14 d, *n* = 451	Staged infant formula with bMFGM,^9^ 60 mg/100 mL bLf, 480–558 mg/d	Exclusive formula feeding until 120 d, stage 1 formula up to 180 d old, stage 2 formula up to 365 d old	Staged infant formula (per intervention protocol)	12 mo	At 18 months:↓ Respiratory illness^10^ incidence^4^↓ URTI incidence^4^↓ Cough incidence^4^↓ Respiratory illness^10^ episode frequency^4^↓ Number of subjects with >1 URTI episode^4^
Motoki, 2020 (Japan) (52)	Healthy children, 12–32 mo, *n* = 101	Growing-up formula with bLf, 48 mg/d	1× sachet with 48 mg bLf, once daily, NFD	Growing-up formula (1x sachet) once daily	13 wk	Respiratory illness^11^↔ Incidence↓ Cumulative duration^4^↔ Episode duration↔ Medication use
Yen, 2011 (Taiwan) (54)	Healthy children, 2–6 y, *n* = 172	Growing-up formula with bLf 35 mg/100mL, 70–85 mg/d	200–240mL of formula, once daily, morning	Growing-up formula, 200–240 mL, once daily, morning	15 mo	↔ Incidence of RTIs (sinusitis, GAS pharyngitis, bronchopneumonia)

1 bLf, bovine lactoferrin; bMFGM, bovine milk fat globule membrane; CFU, colony forming units; FOS, fructo-oligosaccharide; GAS, group A streptococcal; GOS, galacto-oligosaccharide; LRTI, lower respiratory tract infection; NFD, not further described; NR, not reported; ref, reference; RTI, respiratory tract infection; URI, upper respiratory illness; URTI, upper respiratory tract infection; w/o, washout; ∼, average intake; ↓, significant decrease, ↑, significant increase, ↔, no change.

2 Includes a sore throat, cough, nasal secretion, nasal congestion, headache, chills, and fatigue in diary records checked by principal investigator.

3 Decreased in 600mg bLf compared to placebo only.

4 Significant difference between groups in change compared with baseline.

5 Defined as any 2 self-reported cold-associated symptoms, including sore throat, nasal congestion, sinus swelling, sneezing, cough, headache, and fatigue that persisted and lasted 2 d or more.

6 Respiratory-related illnesses (including at least rhinorrhea, cough, wheezing, or nasal congestion).

7 Rhinorrhoea, cough, sore throat, or conjunctivitis for 2 consecutive days increased from baseline.

8 Clinician-confirmed alteration in respiratory status as manifested by chest retractions, tachypnea, rales, wheezing, barky cough or stridor, or an abnormal chest radiograph.

9 Infant formula with bMFGM and added whey protein lipid concentrate (5 g/L).

10 Includes URTI and cough.

11 Includes more than 1 symptom of nasal secretion/congestion, cough/sputum, fever (≥38.0°C) or fatigue.

### Effect of Lf supplementation on markers of systemic inflammation

Characteristics of the 13 trials which reported inflammatory biomarker outcomes following bLf supplementation are presented in Table 1. Most trials were performed in adults (*n* = 12), with 7 trials in females only, 4 trials in both males and females, and 1 trial in males only. These trials were mostly performed in subjects with inflammatory conditions, including pregnant and nonpregnant women with HT (*n* = 2) and ID (*n* = 2), AD, T2D, PCOS, and AK and in bedridden subjects with neurological disease. One trial in healthy children was also included. Trials used bLf interventions, in doses ranging from 32.4 mg to 1000 mg/d (median: 200 mg/d), for a minimum of 3 and maximum of 60 wk (median: 10 wk). Most trials (*n* = 8, 62%) reported a significant improvement in at least 1 systemic inflammatory biomarker, including reduced CRP, TNF-α, IFN-γ, IL-1β, and IL-6, and increased IL-10 and IL-12+p40.

Circulating CRP concentrations were measured in 6 trials, of which 2 trials (33%) reported a decrease and 4 trials reported no change or difference between bLf and control groups. CRP decreased following 12 wk of treatment with whey protein isolate (WPI) powder and bLf 32.4 mg/d in adults with T2D (55) and myo-inositol powder with bLf 200 mg/d in females with PCOS (59), although other trials using bLf 250 mg for 56 d in AD (41) and 180 d in healthy females (40) and dosages of 1000 mg/d for 12 wk in neurological patients or 63 d in healthy females found no effect.

Circulating TNF-α concentrations were measured in 3 trials, of which 2 trials (66%) reported a significant decrease and 1 trial (33%) reported no change. Decreased TNF-α was reported following bLf 250 mg/d for 180 d in healthy females (41), and WPI powder with bLf 32.4 mg/d for 3 mo in adults with T2D (55), whereas there was no difference in TNF-α following bLf 1000 mg/d for 12 wk in neurological patients (53).

Serum IFN-γ concentrations were measured in 2 trials, of which 1 trial (50%) reported a significant decrease following bLf 250 mg/d for 180 d in healthy females (41) and 1 trial (50%) reported no change following infant formula with bLf ∼70 mg/d for 15 mo in young children (54).

Serum IL-1β concentrations were measured in 2 trials, of which 1 trial (50%) reported a significant decrease following bLf 250 mg/d for 180 d in healthy females (41) and 1 trial (50%) reported no change following 1000 mg/d for 63 d in healthy females (48).

Circulating IL-6 concentrations were measured in 7 trials (41, 43, 45–48, 55), of which 6 trials (86%) reported a significant decrease following bLf supplementation. These trials used a range of bLf dosages and durations from 32.4 mg/d for 3 mo (41), 200 mg/d for ≥30 d (43, 45–47), and 250 mg/d for 180 d (41). The subject population of these trials included healthy females (41), adults with T2D (55), pregnant females with ID (45, 46) or HT (43, 47), and nonpregnant females with HT (43). IL-6 concentrations did not change in the nonpregnant female group with ID (46) following 200 mg/d for 30 d, or in healthy females supplemented with 1000 mg/d for 63 d (48).

Circulating IL-10 concentrations were measured in 3 trials, of which 1 (33%) reported a significant increase following bLf 32.4 mg/d for 3 mo in adults with T2D (55), although there was no change following 1000 mg/d for 63 d in healthy females or infant formula with bLf ∼70 mg/d for 15 mo in young children (54).

Serum IgE was measured in 2 trials, and did not change following either bLf 400 mg/d for 12 wk in subjects with AK (35) or 250 mg for 56 d in participants with AD (41).

Other inflammatory biomarkers that were not affected by bLf supplementation included serum eosinophil cationic protein (ECP) (35) after 400 mg/d for 12 wk in subjects with AK and plasma IL-16 and IL-18 after 150 mg/d for 21 d in male athletes (57).

### Dose of Lf required to reduce systemic inflammation

In adults, inflammation (IL-6) was attenuated when Lf was administered alone in doses of 200 mg/d in pregnant and nonpregnant females with iron homeostasis disorders (43, 45–47), although doses of only 32 mg/d given in combination with whey protein were also effective in reducing CRP, TNF-α, and IL-6 in T2D (55). Lf doses >200 mg/d, given either alone or in combination with other compounds, were effective in reducing systemic inflammation outcomes in some (41), but not all (35, 40, 48, 53), trials. Doses <200 mg/d were not effective in some combinations in adults (57) and children (54).

### Meta-analyses for the effect of Lf supplementation on markers of systemic inflammation

**FIGURE 2:**
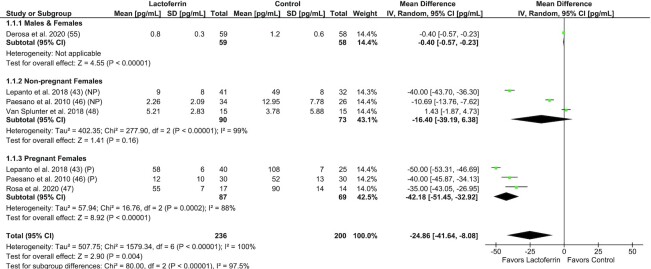
Forest plot of trials investigating the effect of lactoferrin supplementation on circulating IL-6 concentration in adults, subgrouped by sex and pregnancy status. Individual trial effect estimates (boxes) and pooled effect estimate (diamond) for IL-6 are shown. Values are mean differences with 95% CIs determined with the use of generic IV random-effects models. Heterogeneity was quantified by *I*^2^ at a significance of *P* < 0.10. IV, inverse variance; NP, nonpregnant; P, pregnant.

**FIGURE 3:**
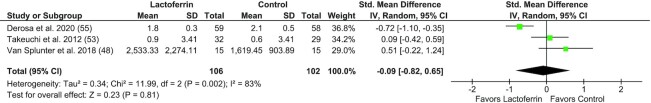
Forest plot of randomized controlled trials investigating the effect of lactoferrin supplementation on circulating CRP concentration in adults. Individual trial effect estimates (boxes) and pooled effect estimate (diamond) for CRP are shown. Values are standardized mean differences with 95% CIs determined with the use of generic IV random-effects models. Heterogeneity was quantified by *I*^2^ at a significance of *P* < 0.10. CRP, C-reactive protein; IV, inverse variance; Std., standardized.

Meta-analysis was performed to examine the effect of Lf supplementation on IL-6 in adults (*n* = 5 trials, *n* = 436 subjects), including 3 trials performed in pregnant/nonpregnant females with ID (46) or HT (43, 47) (**Figure 2**). Lf was associated with a significant reduction in IL-6 compared with control (MD: –24.9 pg/mL; 95% CI: –41.6, –8.1 pg/mL; *I*^2^ = 100%; *P* = 0.004). Subgroup analyses by sex and pregnancy status showed that Lf reduced IL-6 in males and females (*P* < 0.01) and pregnant females (*I*^2^ = 88%; *P* < 0.01), but not in nonpregnant females (*I*^2^ = 99%; *P* = 0.16). Two trials were unable to be included in the IL-6 meta-analysis due to either unusable summary statistics (41) or no control group (45). Meta-analysis was performed to examine the effect of Lf supplementation on CRP in adults (*n* = 3 trials, *n* = 208 subjects), including trials in healthy older females, T2D, and bedridden subjects with neurological disease (**Figure 3**). Lf supplementation was not associated with a change in circulating CRP compared with control (SMD: –0.09; 95% CI: –0.82, 0.65; *I*^2^ = 83%; *P* = 0.81), although 3 trials were unable to be included due to unusable summary statistics (41), no control group (59), or data not published and unable to be obtained from authors (40). Meta-analyses for other systemic inflammatory biomarkers were not performed due to small study numbers and limited available data.

### Effect of Lf supplementation on markers of immune function

Characteristics of the 8 studies examining the effect of Lf supplementation on peripheral immune cell populations and NK cell activity are described in Table 2 (36–39, 42, 44, 48, 53). All trials used bLf supplementation as the intervention, with intervention doses ranging from 100 mg/d to 3 g/d (median: 450 mg/d), and the intervention period ranged from 7 d to 12 mo (median: 8 wk). All trials were conducted in adults, with trials in healthy subjects (*n* = 5, 63%) and 1 trial each in periodontal disease, patients with colonic polyps, and bedridden subjects with neurological disease. Six out of 8 (75%) trials reported on the effect of bLf on peripheral immune cell populations (36–38, 42, 44, 48), while 5 out of 8 (63%) trials reported on changes to immune cell activity following Lf intervention (37–39, 44, 53).

Two out of 6 (33%) trials reported a change in the proportion of ≥1 immune cell population (37, 44). Kawakami et al. (37) reported an increase in the proportion of CD16+, CD56+, and CD86+ cells in healthy, older adults following 3 mo of 300 mg/d enteric-coated bLf supplementation. This same trial also reported an increase in NK cell activity. However, Kozu et al. (38) found no change in the proportion of CD16+ and CD56+ cells in peripheral blood following 1.5 g/d or 3 g/d bLf supplementation for 12 mo in subjects with colonic polyps (38). Mulder et al. (44) reported no change in the proportion of CD3+, CD4+, and CD8+ cells, yet saw changes in activation markers, with an increase in the proportion of CD69-positive CD3+, CD4+, and CD8+ cells, following 7 d of supplementation with 200 mg bLf, compared with baseline. Examining the duration of these trials and the effects seen, changes in activation markers were seen following 7-d supplementation (44); however, changes to peripheral immune cell populations were observed only following 3-mo supplementation (37).

Two out of 5 (40%) trials reported an increase in NK cell activity following bLf intervention (37, 38). Evidence supporting a role of bLf in increasing NK cell activity was primarily seen in trials in older, adult populations. Trials reporting an increase in NK cell activity following Lf supplementation used intervention dosages of 300 mg/d (37) and 1.5 g/d (38), with 300-mg/d delivery via enteric-coated capsules, supplementing subjects for 3 and 12 mo, respectively. Negative trials used 200 mg/d (44), 600 mg/d (52), and ∼1 g/d (53), with supplementation periods of 7 d, 12 wk, and 12 wk, respectively. All 3 trials reporting on the effect of bLf supplementation on neutrophil phagocytic capacity found no difference between bLf and controls (37, 39, 53). These trials were all 12 wk in duration, using bLf dosages from 200 mg to 1 g/d. One trial reported a decrease in neutrophil sterilizing activity following a 12-wk intervention with an enteral formula containing 1 g/L bLf (53).

Two studies performed ex vivo cell culture experiments following in vivo Lf supplementation in healthy, adult populations, which both reported significant changes in response to treatment. Following 4 wk of supplementation with bLf 180 mg/d, Ishikado et al. (36) reported a decrease in TNF-α, IL-1β, and IL-6 production from peripheral blood mononuclear cells (PBMCs) in response to Toll-like receptor (TLR) 4 (TLR4) stimulation via LPS. Van Splunter et al. (48) reported that following bLf 1000 mg/d for 63 d in healthy females, upon TLR7/8 stimulation of PBMCs the percentage of IL-6 and TNF-α–positive plasmacytoid dendritic cells (pDCs) increased.

### Dose of Lf required to modulate immune function

Changes in the proportion of peripheral immune cell populations, and/or their markers of activation, were seen with Lf doses starting from 200 mg/d (44) and 300 mg/d (37), although trials using higher Lf doses also reported no effect (38, 48). One trial reported increased NK cell activity with an Lf dose >1000 mg/d (38); however, in other trials, 100 mg/d (44), 200 mg/d (39, 44), or 600 mg/d (39) bLF alone or 1000 mg/d in enteral formula (53) did not affect NK cell activity. Beneficial changes in ex vivo immune responses were seen with Lf doses as low as 180 mg/d (36), although also with higher doses (1000 mg/d) (48).

### Meta-analyses for the effect of Lf supplementation on markers of immune function

**FIGURE 4:**
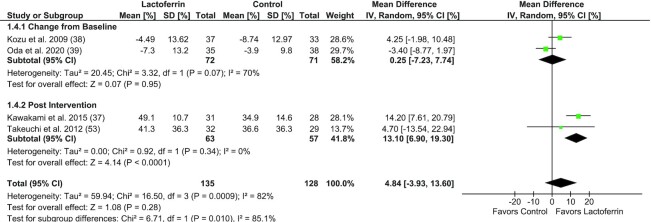
Forest plot of randomized controlled trials investigating the effect of lactoferrin supplementation on NK cell cytotoxicity in adults, subgrouped by outcome variable type (change from baseline or postintervention values). Individual trial effect estimates (boxes) and pooled effect estimate (diamond) for NK cell cytotoxicity are shown. Values are mean differences with 95% CIs determined with the use of generic IV random-effects models. Heterogeneity was quantified by *I*^2^ at a significance of *P* < 0.10. IV, inverse variance.

Meta-analysis was performed to examine the effect of Lf supplementation on NK cell cytotoxicity in adults (*n* = 4 trials, *n* = 280 subjects), including trials in healthy subjects, subjects with colonic polyps, and bedridden subjects with neurological disease (**Figure 4**). There was no difference between Lf-supplemented groups compared with control (MD: 4.84%; 95% CI: –3.93%, 13.60%; *I*^2^ = 82%; *P* = 0.28). One trial was unable to be included in meta-analysis, which was a nonrandomized crossover trial with no washout period, which found no change in NK cell activity following 7 d of Lf supplementation with either 100 mg/d or 200 mg/d (44).

### Effect of Lf supplementation on the incidence, duration, and severity of RTIs

Characteristics of the 10 trials that reported on the incidence, frequency, duration, and severity of RTI outcomes following bLf supplementation are presented in Table 3. Trials were performed in healthy infants and young children (*n* = 5, 50%), healthy adults (*n* = 4, 40%), and in bedridden neurological patients (*n* = 1, 10%). Interventions were mostly bLf-fortified infant or enteral formula (*n* = 6, 60%), with doses ranging from 35 mg/d to 1000 mg/d (median: 350 mg/d) and durations from 4 wk to 15 mo (median: 12 wk). Six of the 10 trials (60%) reported a decrease in at least 1 RTI outcome following bLf supplementation, compared with control, while 4 trials found no effect of bLf supplementation.

Nine trials reported on the incidence of respiratory illness or RTIs, of which the majority (39, 50, 52–54, 58) (*n* = 6, 67%) reported no difference between bLf-supplemented groups and controls, with only 3 trials (33%) reporting a decreased incidence of either respiratory illness or RTIs. During 3-mo feeding of ∼35.8 mg/d bLf-fortified infant formula in healthy infants, Chen et al. (49) reported a decreased incidence of overall respiratory-related illness, which included at least rhinorrhea, cough, wheezing, or nasal congestion, and decreased incidence of rhinorrhea, cough, and wheezing symptoms—although there was no effect on the incidence of nasal congestion. In a 12-mo intervention of bLf-fortified infant formula with a dose of 833 mg/d in healthy infants, King et al. (50) reported a decreased incidence of lower RTIs (LRTIs), although there was no difference in upper RTI (URTI) incidence compared with controls who were given a bLf-fortified infant formula with a dose of 100 mg/d. At 18 mo, following a 12-mo intervention of bLf-fortified infant formula with a dose of 480–558 mg/d in healthy infants, Li et al. (51) reported a decreased incidence of respiratory illness, including both cough and URTIs.

Four trials reported on respiratory illness/RTI frequency, of which 2 trials (51, 56) (50%) reported a decrease in bLf-supplemented subjects compared with controls and 2 trials (39, 58) (50%) reported no difference. Both the frequency of URTIs and cough episodes per subject and the number of subjects with >1 episode were decreased in healthy infants using 480–558 mg/d bLf-fortified infant formula for 12 mo compared with a control formula (51). In adults, the frequency of cold episodes decreased in healthy subjects using a whey protein supplement with 400 mg/d bLf for 90 d compared with control (56). However, there was no effect on the episode frequency of summer colds with either 200 mg or 600 mg/d bLf for 12 wk in another trial in healthy adults (39), or in the frequency of URTIs, colds, or influenza-like illness with synbiotic powder fortified with bLf 300 mg/d for 90 d in healthy males (58).

Six trials reported on either the cumulative duration (total days affected) or the average episode duration of respiratory illnesses or RTIs, with 3 trials (39, 49, 52) (50%) reporting a decrease and 3 trials (50, 56, 58) reporting no difference in bLf-supplemented subjects compared with controls. A 3-mo intervention of ∼35.8 mg/d bLf-fortified infant formula in healthy infants reported a decreased duration of runny nose episodes, but no effect on the duration of wheezing or cough episodes (49), while there was a decrease in the cumulative duration of respiratory illness, but no effect on the episode duration in healthy children given 48 mg/d bLf-fortified formula for 13 wk (52). There was no difference in the cumulative duration of either URTIs or LRTIs in healthy infants given bLf-fortified infant formula with a dose of 833 mg/d for 12 mo or in colds reported in healthy adults using a whey protein supplement with 400 mg/d bLf for 90 d. In healthy adults, Oda et al. (39) reported decreased episode duration of summer colds following 600 mg/d bLf for 12 wk compared with placebo. However, Pregliasco et al. (58) reported no difference in the episode duration of URTIs, colds, or influenza-like illness with synbiotic powder fortified with bLf 300 mg/d for 90 d in healthy males.

Illness severity was reported in 2 trials (56, 58), of which both trials (100%) reported no effect of bLf supplementation compared with controls. There was no effect on the severity of URTI, colds, or influenza-like illness episodes with synbiotic powder fortified with bLf 300 mg/d for 90 d in healthy males (58), and no effect on the severity of cold episodes in healthy adults using a whey protein supplement with 400 mg/d bLf for 90 d (56).

In terms of negative findings, Dix and Wright (42) reported no effect of either microencapsulated or standard bLf capsules at 200 mg/d or 600 mg/d for 4 wk on viral infections (not further described) in healthy males; however, this crossover trial had only 3 subjects per group, no untreated/placebo group, and was limited by the 2 × 4-wk intervention durations. In a trial using a synbiotic powder fortified with bLf 300 mg/d for 90 d in healthy males, Pregliasco et al. (58) reported no effect of bLf on the incidence, episode frequency and duration, or severity of respiratory illnesses compared with control (synbiotic). There was also no difference in RTI incidence between enteral formula and bLf 1000 mg/d fortified enteral formula for 12 wk in bedridden neurological patients. Yen et al. (54) also reported no difference in the incidence of RTIs between infant formula and bLf 70–85 mg/d fortified infant formula for 15 mo in healthy young children.

### Dose of Lf required to prevent RTIs

In adults, bLf 400 mg/d reduced the frequency of cold episodes (56) and 600 mg/d reduced the duration of summer colds (39). However, trials using lower bLf doses given alone [200 mg/d (39)], lower doses in combination with synbiotic [300 mg/d (58)], and higher doses in enteral formula [1000 mg/d (53)] showed no effect on RTI outcomes. In children, formula supplemented with bLf doses as low as 35 mg/d reduced both incidence and duration of respiratory illness in infants (49), although other trials using <100 mg/d reported either no effect on RTI incidence in older children (54) or only a reduction in the cumulative duration, not incidence, of RTIs (52). Higher bLf concentrations in infant formula, including 480–558 mg/d, were associated with reduced RTI illness and frequency in 1 trial (51), although in another trial using formula with 833 mg/d, only LRTI incidence was reduced, while URTI incidence and duration were no different from the control formula, which also contained bLF 100 mg/d (50).

### Meta-analyses for the effect of Lf supplementation on RTI incidence

**FIGURE 5:**
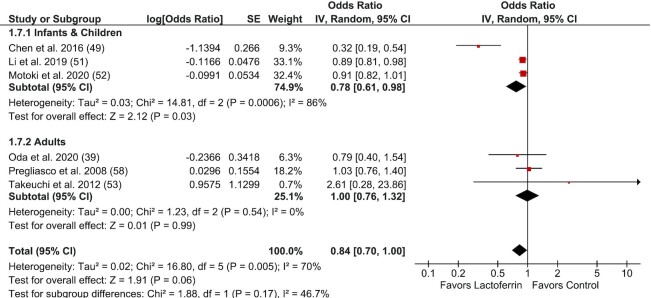
Forest plot of randomized controlled trials investigating the effect of lactoferrin supplementation on incidence of respiratory tract infection/respiratory illness in subjects of all ages, subgrouped by life stage. Individual trial effect estimates (boxes) and pooled effect estimate (diamond) for respiratory tract infection/respiratory illness are shown. Values are ORs with 95% CIs determined with the use of generic IV random-effects models. Heterogeneity was quantified by *I*^2^ at a significance of *P* < 0.10. IV, inverse variance.

Meta-analysis was performed to examine the effect of Lf supplementation on RTI incidence (*n* = 5) (**Figure 5**). Overall, Lf was not associated with a reduction in RTI incidence compared with control, although a trend towards significance was evident (OR: 0.84; 95% CI: 0.70, 1.00; *I*^2^ = 70%; *P* = 0.06). Subgroup analyses by life stage showed Lf reduced RTIs in infants and children (OR: 0.78; 95% CI: 0.61, 0.98; *P* = 0.03), although there was significant heterogeneity identified in this subgroup (*I*^2^ = 86%; *P* < 0.01). In adults, there was no reduction in RTIs (OR: 1.00; 95% CI: 0.76, 1.32; *I*^2^ = 0%; *P* = 0.99). Three trials were unable to be included in this meta-analysis due to unusable summary statistics (50, 54, 56) and 1 trial that was a nonrandomized crossover trial with no washout period that did not provide data on RTI incidence (42).

## Discussion

This systematic literature review examined the effect of Lf supplementation on systemic inflammation, immune function, and RTIs in healthy adults and children as well as adults with various inflammatory conditions. The review presents evidence supporting the role of bLf in reducing systemic inflammation, specifically IL-6. However, evidence for the effect of bLf on improving immune function and preventing RTIs is less clear. While potential benefits have been identified in this review, further research is required to explore the effect of Lf supplementation on immune function. Evidence for Lf providing a protective role in RTIs has been identified in infants and in children, although evidence in adults is limited.

There was a significant effect of bLf reducing IL-6 concentrations in specific subject populations and encouraging evidence for reduced TNF-α; however. effects on other inflammatory biomarkers, including CRP, IFN-γ, IL-1β, IL-10, and IgE, were reported less often, with positive effects seen in ≤50% of trials. The qualitative assessment of evidence was supported by findings from meta-analyses, which found IL-6 was reduced following Lf supplementation; however, there was no effect on CRP concentrations. The trials included in this review that reported reduced IL-6 with bLf supplementation were primarily in female subjects (6 of 7 trials) and mostly in populations with iron homeostasis disorders such as ID and HT (4 of 7). The effect of bLf on IL-6 may be due to several mechanisms, although the role of bLf in regulating iron homeostasis appears to be most relevant to the evidence presented here. Specifically, ferroportin, which is responsible for transporting iron from tissues to the systemic circulation, is downregulated by IL-6, while hepcidin inhibits iron transport and is upregulated by IL-6 (60). bLf treatment both restores iron homeostasis and reduces inflammation by lowering IL-6 and hepcidin, and upregulating ferroportin (43, 61). Various in vitro models have shown the efficacy of bLf in attenuating infection-driven inflammation in epithelial cell models and in reducing IL-6 in LPS-stimulated macrophages (60). bLf has also been shown to downregulate inflammatory cytokine production from epithelial cells in the context of chlamydia infection and cystic fibrosis models (62), although differentially increases inflammatory responses in intestinal epithelial models by increasing IL-8 production, thereby recruiting neutrophils to infection sites, which may eliminate the infection (62). Even though decreased markers of systemic inflammation were seen in 1 trial with healthy, older females, the evidence reviewed here, along with mechanistic studies, suggests that Lf may be most effective in reducing inflammation associated with disease, infection, or in cases of disrupted iron homeostasis.

Less than half of the included trials reported beneficial changes in immune cell phenotype or immune cell function. Immune cell phenotype was assessed via changes in the proportion of peripheral immune cell populations, and/or their markers of activation. Mulder et al. (44) reported no change in NK cell number following 7-d bLf supplementation, Kawakami et al. (37) reported an increase in both CD16^+^ and CD56^+^ lymphocytes following 3-mo 300-mg/d bLf supplementation. NK cells are defined as CD3^–^CD56^+^ lymphocytes, with subsets of CD56^dim^ and CD56^bright^ cells (63). The former subset, in combination with CD16 expression, generally exhibit high cytotoxic capacity. Whether there was a change in the subsets of NK cells in the Lf trials included here is unclear and may be an important consideration in future trials. Markers of immune function examined included NK cell activity, neutrophil functional assays, and ex vivo stimulation of peripheral immune cells. All 3 studies examining neutrophil function reported no change in neutrophil phagocytic capacity with Lf supplementation (37, 39, 53). There was inconsistent evidence for the effect of bLf supplementation on NK cell activity. Approximately half of included trials reported an increase in NK cell activity following bLf supplementation, while the remaining studies reported no effect. Indeed, meta-analysis showed no difference in NK cell cytotoxicity with bLf supplementation compared with control. Studies that found a positive effect of bLf supplementation on NK cell activity were conducted in adults over 40 y of age, while negative trials included subjects in a wide range of ages between 20 and 95 y. This suggests the effect of bLf on NK cell activity may be most effective in an older cohort. Research has shown decreased NK cell activity in older adults is associated with increased incidence and severity of viral infections (64). Thus, whether bLf supplementation increases NK cell activity in older adults, and if it is protective against viral infection, warrants further investigation.

There was a limited number of trials examining the effect of Lf supplementation on ex vivo cell cultures. Ishikado et al. (36) reported a decrease in proinflammatory cytokine release in response to LPS stimulation by PBMCs isolated from subjects with periodontal disease, following 4-wk supplementation with 180-mg/d bLf. Immune cells found within PBMCs are responsible for inflammatory responses to LPS from gram-negative bacteria, which form plaque on the subgingival tissue, with elevated inflammatory responses associated with periodontal disease severity (65). Thus, in the context of this disease, a reduced inflammatory response from PBMCs may be perceived as beneficial. Van Splunter et al. (48) reported that, upon TLR7/8 stimulation, Lf supplementation increased intracellular IL-6 and TNF-α production in pDCs in elderly women. Previous studies have reported a significant role of pDCs in the antiviral (IFN-α) response elicited by the PBMC population following viral infection (66). Further, pDCs may produce a moderate amount of proinflammatory cytokines, such as IL-6 and TNF-α. Aging is associated with immune dysfunction, with data demonstrating declining responses to TLR stimulation from dendritic cells (67, 68). Hence, these data may suggest that bLf supplementation restores these responses. While data from these 2 studies suggest a beneficial impact of Lf supplementation, further research is needed to evaluate effects on immunity in the context of infection, such as respiratory viruses, and a variety of hosts.

The evidence presented here for the effect of Lf supplementation on immune function is heterogeneous. However, this is likely due to a lack of research, small sample sizes, and variability in dose, duration, and delivery mode of Lf. No research fitting our inclusion criteria examined the effect of Lf supplementation on immune function in children. Furthermore, studies investigating the effect on immune function used all bLf supplementation, with no studies examining the effect of rhLf. Future research should aim to clarify the effect of different types, dosages, and delivery of Lf supplementation on immune function, in adequately powered trials, in both adults and children.

The summary of evidence for the effect of bLf supplementation on either the incidence, duration, or severity of respiratory illness and RTIs included 10 studies, which reported reductions in RTI incidence in 33% of trials and reductions in frequency and/or duration in 50% of trials, while no trials reported a reduction in illness severity. Three out of the 5 (60%) trials in children and 2 out of the 5 (40%) trials in adults reported benefits associated with bLf supplementation compared with the control treatment, with intervention durations of at least 3 mo and dosages from 35 mg/d to 833 mg/d in infants and 400 mg/d to 600 mg/d in adults. Meta-analysis results indicated that Lf supplementation was associated with reduced RTI incidence in infants and children, but not in adults. A recently published meta-analysis including RCTs using Lf for the prevention of RTI occurrence reported a reduction in RTIs with bLf supplementation compared with control (OR: 0.57; 95% CI: 0.44, 0.74; *n* =1194) (69). This meta-analysis included 6 studies, 4 of which were included in the RTI meta-analysis presented here, with 1 study in adults and 5 in infants, including preterm infants. The outcomes of the 2 meta-analyses are different, due to the differing review inclusion criteria and analysis methodology.

RTIs are primarily caused by bacteria and viruses; thus, Lf supplementation for the prevention of RTIs has been suggested due to the broad-spectrum antibacterial, antiviral, and immunomodulating functions of Lf. Antibacterial actions are related to direct effects of Lf binding iron, which impedes the growth of micro-organisms, and through iron-independent mechanisms including direct effects on gram-negative bacteria by binding LPS and preventing bacterial adhesion and entry to host cells (2, 70). Antiviral effects are related to direct effects of Lf binding and blocking viral receptors (heparan sulfate proteoglycans) (14), reducing viral replication by inducing type I IFN production (5), and indirect effects on immune cells, such as increased phagocytic activity of macrophages (71) and enhanced NK cell activity (5). While mechanistic studies support the antiviral and antibacterial capabilities of endogenous Lf, in agreement with the evidence reviewed here on the effect of bLf on immune function, evidence from human in vivo supplementation trials with bLf for the prevention or amelioration of RTIs is limited. This discrepancy may be due to either the formulation, dosage, or duration of supplementation used in intervention trials, or the population being studied. The lack of effect seen on RTIs in humans has also been seen in murine models of bLf administration, where no effect was seen on viral loads or inflammation in both respiratory syncytial virus (72, 73) and influenza infection models. Contradictory reports on the efficacy of bLf in modulating the response to RTIs in experimental models may be due to the type of pathogen involved, as protective effects of bLf have not been seen in all experimental models (74). This is supported by the results seen in King et al. (50) where, in healthy infants supplemented with bLf (833 mg/d)-enriched formula, the incidence of LRTIs decreased, but there was no difference in URTI incidence between the intervention and control group. This trial may highlight the effect of Lf on different RTI pathogens, as LRTIs and URTIs are associated with different viral and bacterial etiologies (75). The body of evidence in this review suggests that Lf supplementation may play a role in preventing RTIs in healthy children. Another systematic review found that Lf prevents late-onset sepsis, necrotizing enterocolitis, and hospital-acquired infection in preterm infants (17), suggesting that Lf supplementation is also beneficial in populations with impaired or immature immune systems.

The summary of evidence was examined to assess whether Lf-induced improvements in inflammation or immune function occurred in tandem or were associated with decreased RTIs. Two trials reported both the effect of bLf on NK cell activity and RTIs, with both trials finding no change in NK cell activity and the incidence of summer colds (39) or RTIs and CRP concentrations (53), although Oda et al. (39) did report decreased duration of summer cold episodes following bLf 600 mg/d for 12 wk. While some changes in the antiviral response of TLR7/8-stimulated pDCs were seen with bLf intervention, including increased intracellular production of IL-6 and TNF-α, there was no effect on a comprehensive suite of systemic inflammatory biomarkers following bLf 1 g/d in healthy, older females (48). Further, following a 15-mo trial of ∼70 mg/d bLf formula, there was neither a change in inflammatory biomarkers (IFN-γ, IL-10) nor RTIs in healthy children (54). As multiple outcomes were seldom reported in the studies included in this review, it is not possible to determine whether beneficial changes in inflammation or immune function are related to reduced RTI illness.

The mode of administration may be important for the effects of bLf on inflammation, as suggested by Rosa et al. (47), where IL-6 levels were reduced in HT pregnant females only when bLf was taken before, and not during, meals. This finding was supplemented by an experiment showing that bLf was almost completely digested in the presence of gastric juices collected after meals, in contrast to the partial degradation seen when exposed to gastric juices sampled before meals. This report suggests that the digested peptides of bLf are ineffective in reducing inflammation, which agrees with most trials in this review that showed a decrease in inflammation when bLf was administered before meals. Most trials that gave bLf either with meals, in a feeding formula, or did not specify the timing of administration in relation to meals did not report a reduction in systemic inflammatory biomarkers. The exceptions were Bharadwaj et al. (41), which delivered a milk ribonuclease-enriched bLf supplement daily with unspecified timing, and Genazzani et al. (59), which delivered a bLf-enriched myoinositol powder supplement twice daily at 10:00 and 16:00 h, which may have been far enough away from meal times to avoid digestion by gastric juices containing higher quantities of proteolytic enzymes. Examining the timing of administration in trials reporting on immune function following bLf supplementation provides unclear results. Beneficial changes in immune cell populations and immune function were seen in trials where bLf was administered both with and after meals, as well as in trials where timing was not specified. The timing of administration also does not appear to modify the effect of bLf in preventing or ameliorating RTIs, as most studies delivered bLf in formula, of which 4 out of 5 reported beneficial effects, while 1 of the 2 studies prescribing bLf after meals and 1 of the 2 studies where timing was not specified also reported reduced frequency or duration of RTIs.

Digestion and absorption of supplemental bLf are important considerations for mode and timing of supplement administration. bLf is absorbed intact by epithelial cells in the small intestine via interaction with intelectin-1 and endocytosis (76), then enters the circulation through the lymphatic pathway (77), in a similar manner to hLf (78). It has been suggested that enteric coating or liposome encapsulation may increase the bioavailability of bLf by reducing the degradation of Lf by pepsin and trypsin in the stomach, allowing greater amounts of intact Lf to be absorbed in the intestine (79). However, other reports suggest that bLf is relatively robust to enzymatic digestion, with over 60–80% of bLf (depending on the level of iron saturation) resisting degradation in the adult GIT in vivo (80). Furthermore, while liposomal bLf resisted digestion to a greater extent, nonliposomal bLf also remained intact after digestion in animal models (81). The physiological roles of bLf are reliant upon the structure of the bLf protein and the level of iron saturation (78). Consequently, the intact protein and absorbed fragments of bLf digestion exert differential effects (82). Gastric hydrolysis yields the biologically active component of Lf located near the N-terminus, called lactoferricin, which has potent antibacterial properties that are unrelated to the iron-binding properties of Lf (83). This may explain why benefits of bLf supplementation were still seen for immune function and RTI outcomes in studies where nonencapsulated forms of bLf were administered, or where bLf was administered close to mealtimes. Further, it is important to note that digestion and absorption of Lf are distinct in infants compared with adults, due to a higher gastric pH, which enables greater absorption of intact Lf (84). This difference may explain why beneficial effects were seen with addition of bLf to infant formula, although generally not with enteral formula in adults. Another mode of administration factor that may influence Lf effects on both innate and adaptive immune responses is whether the oral preparation is taken as a liquid or solid supplement. Differential effects were seen in animal models using either liquid or solid oral preparations, given in bolus or continuous doses or via intragastric gavage (85).

This review aimed to establish the dose of Lf required for beneficial effects on inflammation, immune function, and RTIs; however, the heterogeneity in terms of subject population, intervention outcomes, and trial duration makes definitive conclusions difficult. When Lf was given alone, doses of 200 mg/d reduced inflammation in adults, alterations in immune function were seen with doses of 180 mg/d in adults, while doses of 35 mg/d in children and 600 mg/d in adults were effective in reducing RTIs. However, several trials with higher Lf doses did not see beneficial effects. Lower doses of Lf were beneficial in trials where Lf was provided in combination with other supplements. Doses of as little as 32 mg/d in combination with whey protein reduced inflammation in adults, and immunoglobulin-enriched Lf at 400 mg/d reduced RTI episode frequency in adults. Further research is required to address this aspect of Lf supplementation.

This systematic review has several limitations, primarily regarding the heterogeneity between included studies in relation to the subject population, bLf intervention formulation, dose, and duration as well as outcome variables. The breadth of outcomes reported in this study provides a comprehensive overview of the effects of Lf supplementation on systemic inflammation and immune function. However, meta-analyses of multiple outcomes possibly contributed to type I error. Further, while some studies reported measuring outcomes included in this review, such as IL-6 and CRP, many did not report the actual data if there was no effect of the intervention, which may have led to bias in meta-analyses. Although the authors of all trials were contacted to retrieve summary statistics and unpublished data for use in meta-analyses where necessary, some trials were not able to be included due to either unusable data or study design. This limited the findings of the meta-analyses for CRP and RTIs and precluded performing meta-analyses on other outcomes. Further, the meta-analyses that were performed included only a small number of studies, with small sample sizes. The findings of this review were also limited by the sample sizes of included studies, of which many were likely to have been underpowered to adequately assess the effects of bLf supplementation, especially for the effects on immune function. The summary of evidence was strengthened by the inclusion of positive- and neutral-quality trials, while negative-quality studies were excluded due to high risk of bias. However, this may have limited the potential conclusions of the review, as almost 30% of trials were considered to have high risk of bias and were therefore excluded from the summary of evidence. These studies with high risk of bias would not change the summary of evidence for this review, as generally the evidence provided is consistent with the findings presented in the included studies. Further, most excluded studies would not be suitable for inclusion in meta-analysis due to unreported data and study design.

The use of Lf to modulate inflammation and immune responses is an emerging area of research. For this reason, several complementary review outcomes were addressed, and the review inclusion criteria were broad to capture all relevant Lf supplementation trials. However, there were still limited clinical data available for inclusion in both the summary of evidence and meta-analyses in this review. This may partly explain why findings are inconsistent within each of the review outcomes, as heterogeneity between studies existed. Further, the effects of Lf are not consistent across the different outcomes investigated here, which may be due to the different mechanisms regulating the respective outcomes. For example, the effects of Lf supplementation on inflammation and immune function/RTIs appear to be mediated by distinct pathways, as discussed earlier in the Discussion. Despite these limitations, this is the first review to systematically collate and summarize the evidence for the effect of Lf on inflammation, immune function, and RTIs, which will assist in designing future Lf intervention studies.

Clinical studies on Lf supplementation are limited; however, the available evidence indicates that a daily dose of 200 mg Lf may reduce IL-6 concentrations in some subject populations. Furthermore, consumption of formulas containing 35–833 mg Lf/d may reduce the incidence of RTIs in infants and children, suggesting improved immune function. Due to the small number of trials and heterogeneous study designs, future research is required to determine effects on immune function, optimal supplementation strategies, and identify subject populations most likely to benefit from Lf supplementation. Overall, this review indicates the need for future research, with methodologically sound, adequately powered studies that address the questions on formulation, required dose, and duration of Lf supplementation on comprehensive outcomes in both adults and children from healthy populations and those with chronic disease.

## Supplementary Material

nmac047_Supplemental_File

## Notes

Supported by a Commonwealth of Australia Innovations Connection Project Grant in partnership with Freedom Foods Group Nutritionals Ltd and the University of Newcastle.

Author disclosures: The authors report no conflicts of interest. The sponsor did not play any role in the design, conduct, analysis, or drafting the manuscript for this study. The authors were free to publish the study results; however, agreed to forward a draft of the proposed publication to the sponsor not less than 30 days prior to the proposed date of publication.

Supplemental Tables 1–3 are available from the “Supplementary data” link in the online posting of the article and from the same link in the online table of contents at https://academic.oup.com/advances/.

Abbreviations used: AD, atopic dermatitis; AK, atopic keratoconjunctivitis; BDCA, blood dendritic cell antigen; bLf, bovine lactoferrin; CD, cluster of differentiation; CRP, C-reactive protein; GIT, gastrointestinal tract; hLf, human lactoferrin; HT, hereditary thrombophilia; ID, iron deficiency; ILC, innate lymphoid cell; Lf, lactoferrin; LRTI, lower respiratory tract infection; MD, mean difference; NCT, noncontrolled trial; NRCT, nonrandomized controlled trial; NRCXT, nonrandomized controlled crossover trial; rhLf, recombinant human lactoferrin; PBMC, peripheral blood mononuclear cell; PCOS, polycystic ovarian syndrome; pDC, plasmacytoid dendritic cell; Q, question; RCT, randomized controlled trial; RCXT, randomized controlled crossover trial; RTI, respiratory tract infection; SMD, standardized mean difference; T2D, type 2 diabetes; TLR, Toll-like receptor; URTI, upper respiratory tract infection; WPI, whey protein isolate.
