# Supplementary material for: Effect of Lactoferrin Supplementation on Inflammation, Immune Function, and Prevention of Respiratory Tract Infections in Humans: A Systematic Review and Meta-analysis
Source: Adv Nutr. 2022 Apr 27;13(5):1799–819. doi: 10.1093/advances/nmac047 (PMC9526865; doi:10.1093/advances/nmac047)
Supplement: nmac047_Supplemental_File [file nmac047_supplemental_file.docx]

**Supplementary Table 1. Ovid MEDLINE Search Strategy (15.12.2020)**

| **#** | **Searches** | **Results** |
| --- | --- | --- |
| 1 | "haemophilus influenza".mp. | 701 |
| 2 | Coronavirus/ or coronavirus.mp. or Coronavirus Infections/ | 70130 |
| 3 | picornavirus.mp. or Picornaviridae/ | 3349 |
| 4 | Enterovirus/ or enterovirus.mp. | 18770 |
| 5 | parainfluenza.mp. | 7800 |
| 6 | Streptococcus pneumoniae/ or "streptococcus pneumoni*".mp. | 34316 |
| 7 | lactoferrin.mp. or Lactoferrin/ | 8836 |
| 8 | lactotransferrin.mp. | 298 |
| 9 | talactoferrin.mp. | 44 |
| 10 | inflammation.mp. or Inflammation/ | 547998 |
| 11 | "inflammatory biomarker*".mp. | 5387 |
| 12 | "inflammat* marker".mp. | 3719 |
| 13 | C-Reactive Protein/ or crp.mp. | 73251 |
| 14 | Fibrinogen/ or fibrinogen.mp. | 63633 |
| 15 | "acute phase protein*".mp. or Acute-Phase Proteins/ | 11633 |
| 16 | interleukin*.mp. or exp Interleukins/ | 361485 |
| 17 | "tumor necrosis factor".mp. or Tumor Necrosis Factor-alpha/ or TNF*.mp. | 276936 |
| 18 | TNF-a.mp. | 2264 |
| 19 | NF-kappa B/ or "NF kappa B".mp. or NFK*.mp. | 76000 |
| 20 | e-selectin.mp. or E-Selectin/ | 9146 |
| 21 | icam*.mp. or Intercellular Adhesion Molecule-1/ | 28245 |
| 22 | vcam*.mp. or Vascular Cell Adhesion Molecule-1/ | 13487 |
| 23 | "exhaled nitric oxide".mp. | 4059 |
| 24 | (eNO or Feno).mp. | 3317 |
| 25 | immunity/ or exp immunity, cellular/ or exp immunity, innate/ or immune system/ or exp antibody-producing cells/ or exp antigen-presenting cells/ or exp leukocytes/ or mast cells/ or exp mononuclear phagocyte system/ or phagocytes/ or "immune function".mp. or "immune system".mp. | 1131482 |
| 26 | ("immune cell response*" or "immune response*").mp. | 253321 |
| 27 | interferon*.mp. or exp Interferons/ or "ifn b*".mp. or IFN*.mp. | 243251 |
| 28 | leukocyte*.mp. or exp Leukocytes/ | 861397 |
| 29 | "white blood cell*".mp. | 33863 |
| 30 | "mast cell*".mp. or Mast Cells/ | 47967 |
| 31 | macrophage*.mp. or Macrophages/ | 308370 |
| 32 | granulocytes/ or basophils/ or eosinophils/ or neutrophils/ or leukocytes, mononuclear/ or granulocyte*.mp. or basophil*.mp. or eosinophil*.mp. or neutrophil*.mp. or leukocyte*.mp. | 539346 |
| 33 | "peripheral blood mononuclear cell*".mp. | 48154 |
| 34 | PBMC*.mp. | 33971 |
| 35 | "b cell*".mp. or B-Lymphocytes/ | 221068 |
| 36 | T-Lymphocytes/ or T-Lymphocytes, Regulatory/ or "T reg* cell*".mp. | 193273 |
| 37 | "NK cell*".mp. or Killer Cells, Natural/ or NK*.mp. | 85886 |
| 38 | "innate lymphoid cell".mp. | 568 |
| 39 | antigen-presenting cells/ or dendritic cells/ or "dendritic cell*".mp. or "antigen present*".mp. | 105599 |
| 40 | "NK cell activity".mp. | 4037 |
| 41 | Lymphocytes/ or T-Lymphocyte Subsets/ or lymphocyte*.mp. | 653303 |
| 42 | exp t-lymphocytes/ or cd4-positive t-lymphocytes/ or cd8-positive t-lymphocytes/ or natural killer t-cells/ or precursor cells, t-lymphoid/ or t-lymphocyte subsets/ | 331507 |
| 43 | "interferon gamma".mp. or Interferon-gamma/ | 93034 |
| 44 | Chemokine CXCL10/ or CXCL10.mp. | 6543 |
| 45 | IP-10.mp. | 4566 |
| 46 | exp respiratory tract infections/ or bronchitis/ or bronchiolitis/ or exp common cold/ or exp influenza, human/ or laryngitis/ or pharyngitis/ or nasopharyngitis/ or tonsillitis/ or pneumonia/ or pneumonia, bacterial/ or pneumonia, viral/ or rhinitis/ or sinusitis/ or "respiratory tract infection*".mp. or bronchitis.mp. or bronchiolitis.mp. or "common cold".mp. or influenza.mp. or laryngitis.mp. or pharyngitis.mp. or nasopharyngitis.mp. or tonsillitis.mp. or pneumonia.mp. or rhinitis.mp. | 573812 |
| 47 | ("upper respiratory tract infection*" or LRTI or URTI or "lower respiratory tract infection*").mp. | 12836 |
| 48 | exp Influenza A virus/ or "influenza a".mp. or influenza*.mp. | 136310 |
| 49 | "influenza b virus".mp. or exp Influenza B virus/ or "influenza b".mp. | 6329 |
| 50 | "influenza C virus*".mp. or Influenzavirus C/ or "influenza C*".mp. | 4885 |
| 51 | rhinovirus*.mp. or exp Rhinovirus/ | 6484 |
| 52 | Respiratory Syncytial Virus Infections/ or Respiratory Syncytial Viruses/ or "respiratory synctial virus*".mp. or RSV*.mp. | 18058 |
| 53 | respiratory syncytial virus, human/ | 2662 |
| 54 | "common cold".mp. or Common Cold/ | 6273 |
| 55 | 10 or 11 or 12 or 13 or 14 or 15 or 16 or 17 or 18 or 19 or 20 or 21 or 22 or 23 or 24 | 1099981 |
| 56 | 7 or 8 or 9 | 9004 |
| 57 | 1 or 2 or 3 or 4 or 5 or 6 or 25 or 26 or 27 or 28 or 29 or 30 or 31 or 32 or 33 or 34 or 35 or 36 or 37 or 38 or 39 or 40 or 41 or 42 or 43 or 44 or 45 or 46 or 47 or 48 or 49 or 50 or 51 or 52 or 53 or 54 | 2475491 |
| 58 | 55 or 57 | 3080556 |
| 59 | 56 and 58 | 3917 |
| 60 | exp animals/ not humans.sh. | 4762182 |
| 61 | 59 not 60 | 3261 |
| 62 | 59 and "Humans" [Subjects] | 2937 |
| 63 | clinical trial.mp. or exp Clinical Trial/ | 963687 |
| 64 | (treatment or treated).mp. | 5730678 |
| 65 | Dietary Supplements/ or supplement*.mp. | 355802 |
| 66 | randomi?ed trial.mp. | 55745 |
| 67 | (placebo or "randomi?ed controlled trial").mp. or Randomized Controlled Trials as Topic/ or Clinical Trials as Topic/ | 929329 |
| 68 | ("clinical trial" or "clinical trial, phase i" or "clinical trial, phase ii" or clinical trial, phase iii or clinical trial, phase iv or controlled clinical trial or "multicenter study" or "randomized controlled trial").pt. or double-blind method/ or clinical trials as topic/ or clinical trials, phase i as topic/ or clinical trials, phase ii as topic/ or clinical trials, phase iii as topic/ or clinical trials, phase iv as topic/ or controlled clinical trials as topic/ or randomized controlled trials as topic/ or early termination of clinical trials as topic/ or multicenter studies as topic/ or ((randomi?ed adj7 trial*) or (controlled adj3 trial*) or (clinical adj2 trial*) or ((single or doubl* or tripl* or treb*) and (blind* or mask*))).ti,ab,kw. or ("4 arm" or "four arm").ti,ab,kw. | 1683735 |
| 69 | intervention*.mp. | 1085440 |
| 70 | clinical study/ | 3676 |
| 71 | random*.mp. | 1419306 |
| 72 | Case-Control Studies/ or Control Groups/ or Matched-Pair Analysis/ or ((case* adj5 control*) or (case adj3 comparison*) or control group*).ti,ab. | 809035 |
| 73 | (therapeutic or therapy).mp. | 6085056 |
| 74 | control groups/ or cross-over studies/ or double-blind method/ or random allocation/ or single-blind method/ | 313480 |
| 75 | (control group or control or study or group or arm).mp. | 12575925 |
| 76 | trial.mp. | 1222839 |
| 77 | 63 or 64 or 65 or 66 or 67 or 68 or 69 or 70 or 71 or 72 or 73 or 74 or 75 or 76 | 17574355 |
| 78 | 62 and 77 | 1974 |
| 79 | 1 or 2 or 3 or 4 or 5 or 6 or 46 or 47 or 48 or 49 or 50 or 51 or 52 or 53 or 54 | 669270 |
| 80 | 10 or 11 or 12 or 13 or 14 or 15 or 16 or 17 or 18 or 19 or 20 or 21 or 22 or 23 or 24 or 25 or 26 or 27 or 28 or 29 or 30 or 31 or 32 or 33 or 34 or 35 or 36 or 37 or 38 or 39 or 40 or 41 or 42 or 43 or 44 or 45 | 2522767 |
| 81 | 7 or 8 or 9 | 9004 |
| 82 | 80 and 81 | 3754 |
| 83 | 79 and 81 | 351 |
| 84 | 82 or 83 | 3917 |
| 85 | 77 and 84 | 2705 |
| 86 | 85 not (exp animals/ not humans.sh.) | 2206 |

| **Supplementary Table 2. Methodological quality assessment of full text articles as determined by the Academy of Nutrition and Dietetics Quality Criteria Checklist^1^** | | | | | | | | | | | | |
| --- | --- | --- | --- | --- | --- | --- | --- | --- | --- | --- | --- | --- |
| **Article**  Author ,Year, Reference | **Design/Level of evidence**^2^ | **Overall Rating** | **Q1** | **Q2** | **Q3** | **Q4** | **Q5** | **Q6** | **Q7** | **Q8** | **Q9** | **Q10** |
| Bharadwaj, 2010 [1] | RCT/II | Ø | Y | Y | N | Y | N | Y | Y | Y | N | N |
| Chen, 2016 [2] | RCT/II | + | Y | Y | Y | Y | Y | Y | Y | Y | Y | U |
| Derosa, 2020 [3] | RCT/II | Ø | Y | Y | Y | Y | Y | N | Y | Y | N | Y |
| Dix, 2018 [4] | RCXT/II | Ø | Y | Y | NA | Y | Y | Y | N | N | N | N |
| Fujishima, 2020 [5] | RCT/II | Ø | Y | N | U | U | Y | N | N | Y | Y | Y |
| Genazzani, 2014 [6] | NCT/IV | Ø | Y | Y | NA | U | NA | N | Y | Y | N | Y |
| Ishikado, 2004 [7] | NCT/IV / RCT/II | - | N | N | NA | U | Y | N | U | Y | N | U |
| Ishikado, 2010 [8] | NCT/IV | Ø | Y | N | NA | Y | N | Y | Y | Y | N | U |
| Kawakami, 2015 [9] | RCT/II | Ø | Y | N | N | Y | Y | Y | Y | Y | Y | N |
| King, 2007 [10] | RCT/II | Ø | Y | Y | N | Y | Y | Y | Y | Y | Y | N |
| Kozu, 2009 [11] | RCT/II | + | Y | Y | Y | Y | Y | Y | Y | Y | Y | N |
| Lepanto, 2018 [12] | NRCT/III-2 | Ø | Y | Y | U | Y | N | N | Y | Y | Y | Y |
| Li, 2019 [13] | RCT/II | + | Y | Y | Y | Y | Y | Y | Y | Y | Y | N |
| Mohamed, 2018 [14] | RCT/II | - | Y | N | N | U | N | N | Y | N | N | Y |
| Mohamed, 2019 [15] | RCT/II | - | Y | Y | N | U | N | N | Y | N | N | Y |
| Motoki, 2020 [16] | RCT/II | + | Y | Y | Y | Y | Y | Y | Y | N | Y | N |
| Mulder, 2008 [17] | NRXT/III-2 | Ø | Y | Y | NA | N | N | N | Y | N | Y | Y |
| Oda, 2020 [18] | RCT/II | + | Y | Y | Y | Y | Y | Y | Y | Y | Y | N |
| Paesano, 2009 [19] | NCT/IV | - | N | N | NA | U | N | N | Y | U | N | U |
| Paesano, 2010 [20] | RCT/II | Ø | Y | Y | N | Y | N | N | Y | Y | N | N |
| Paesano, 2012 [21] | NCT/IV | Ø | Y | N | NA | Y | Y | N | Y | N | N | N |
| Paesano, 2014 [22] | NRCT | - | Y | Y | N | Y | N | N | Y | N | N | N |
| Pregliasco, 2008 [23] | RCT/II | Ø | Y | U | U | Y | Y | N | Y | N | N | Y |
| Rosa, 2020 [24] | NRCT | Ø | Y | Y | N | Y | N | N | Y | Y | N | Y |
| Saraceno, 2014 [25] | RCT/II | - | N | N | N | Y | N | N | Y | U | Y | Y |
| Shin, 2018 [26] | RCT/II | - | Y | N | N | Y | N | Y | N | Y | N | N |
| Takeuchi, 2012 [27] | RCT/II | Ø | Y | Y | Y | Y | N | Y | N | N | Y | N |
| Tong, 2017 [28] | RCT/II | Ø | Y | N | Y | Y | Y | Y | Y | U | Y | N |
| Van Splunter, 2018 [29] | RCT/II | + | Y | Y | Y | Y | Y | Y | Y | Y | Y | N |
| Vitetta, 2013 [30] | RCT/II | Ø | Y | Y | N | Y | Y | Y | N | N | N | Y |
| West, 2012 [31] | RCT/II | Ø | Y | N | U | Y | U | Y | Y | Y | Y | N |
| Yamauchi, 1998 [32] | NCT/IV | - | N | N | NA | U | U | N | N | U | N | N |
| Yen, 2011 [33] | RCT/II | Ø | N | N | N | Y | Y | Y | N | Y | Y | N |
| Zimecki, 1998[34] | NCT/IV | - | N | N | NA | Y | Y | N | Y | N | N | U |
| Zimecki, 1999[35] | NCT/IV | - | Y | N | U | U | U | N | Y | U | N | U |
| RCT, randomised controlled trial; RCXT, randomised controlled cross-over trial; NRCT, non-randomised controlled trial, NCT, non-controlled trial; +, positive study; Ø, neutral study quality; quality; -, negative study quality; Y, yes; N, no; NA, not applicable to study design; U, unable to determine. Q1, Was the research question clearly stated?; Q2, Was the selection of study subjects/patients free from bias?; Q4, Were study groups comparable?; Q5, Was method of handling withdrawals described?; Q6, Were intervention/therapeutic regimens/exposure factor or procedure and any comparison(s) described in detail? Were intervening factors described?; Q7, Were outcomes clearly defined and the measurements valid and reliable?; Q8, Was the statistical analysis appropriate for the study design and type of outcome indicators?; Q9, Are conclusions supported by results with biases and limitations taken into consideration?; Q10, Is bias due to study’s funding or sponsorship unlikely? ^1^Reference:[36], ^2^Reference: [37] | | | | | | | | | | | | |

| **Supplementary Table 3.** **Summary of excluded negative quality trials examining the effect of lactoferrin on systemic inflammatory biomarkers, immune function and respiratory tract infections in adults and children.** | | | | | | |
| --- | --- | --- | --- | --- | --- | --- |
| **Author, Year (Country)** | **Participants**, age, n | **Intervention,**  daily dose | **Protocol** | **Control,** daily dose | **Duration** | **Effect of intervention on inflammation** |
| **ADULTS** | | | | | | |
| Mohamed, 2019 (Egypt) [15] | Alzheimer’s disease (AD), >65 years, **n=50** | bLf capsule, **250mg/d** | 1× 250mg daily NFD | Standard AD therapy, NFD | 3 months | ↓ IL-6^1^  ↑ IL-10^1^ |
| Paesano, 2009 (Italy) [19] | ID or IDA pregnant women, age NR **n=5** | bLf, NFD | bLf for 30 days followed by control for 30 days, no washout period. | Ferrous sulphate, NFD. | 60 days | ? IL-6^2^ |
| Paesano, 2014 (Italy) [22] | Hereditary thrombophilia pregnant (6-8^th^ wk. gestation) females, 18-40 years, **n=40** | bLf capsule plus heparin 0.3 U/day and aspirin 100mg every 2 days, **200mg/d** | 1 × 100mg twice daily, before meals | Ferrous sulphate (520mg/d), 1 x tablet daily during meals plus heparin 0.3 U/day and aspirin 100mg every 2 days | Until delivery | ↓ IL-6^3^ |
| Saraceno, 2014 (Italy) [25] | Psoriasis, 51.6 years (mean), **n=13** | bLf capsule, **200mg/d** plus 10% bLf ointment applied to psoriatic lesions | 1 x 100mg capsule twice daily, before meals | N/A | 4 weeks | ↔ IL-6  ↔ TNF-α |
| Zimecki, 1998 (Poland) [34] | Healthy adults, 26-59 years, **n=17** | bLf capsule, **40mg/d** red cabbage super oxide dismutase 68 mg/d, vitamin C -  120 mg/d, beta-carotene 0.8 mg/d, vitamin E 14mg/d, selenium yeast 50 μg/d. | 1 x 20mg capsule twice daily, NFD. | N/A | 10 days | ? IL-6^2^  ? TNF-α^2^ |
| **CHILDREN** | | | | | | |
| Mohamed, 2018 (Egypt) [14] | T2DM children, 12-17 years, **n=30** | Camel milk colostrum Lf capsule, **250mg/d** | 1× 250mg Lf daily NFD | N/A | 3 months | ↓ IL-6^3^  ↓ IL-1β^3^  ↓ IL-18^3^ |
| **Author, Year (Country)** | **Participants**, age, n | **Intervention,**  daily dose | **Protocol** | **Control,** daily dose | **Duration** | **Effect of intervention on immune function** |
| **ADULTS** | | | | | | |
| Ishikado, 2004 (Japan) [7] | Healthy males, 30-39 years, **n=10** | Liposomal bLf tablet, **318.6mg/d** | 9 x 35.4mg daily, NFD | Non-liposomal bLf tablet, **318.6mg/d** | 4 weeks | Intervention:  ↑ IFN-α (Virus induced)^3,4^  NK Cell activity:  E/T ratio 40:1 ↓^3^  E/T ratio 20:1 ↓^3^  Control:  ↔ IFN-α (Virus induced)^4^  NK Cell activity:  E/T ratio 40:1 ↔  E/T ratio 20:1 ↔ |
| Yamauchi, 1998 (Japan) [32] | Healthy males, 31-55 years, **n=10** | bLf tablet, **2g/d** | 7 x 150mg twice daily, NFD | N/A | 4 weeks | PMNs:  ? Phagocytic activity^2^  ? CD16^+2^  Lymphocytes:  ? CD16^+^ proportion^2^  ? CD11b^+^/56^+^ proportion^2^ |
| Zimecki, 1998 (Poland) [34] | Healthy adults, 26-59 years, **n=17** | bLf capsule, **40mg/d** red cabbage super oxide dismutase 68 mg/d, vitamin C -  120 mg/d, beta-carotene 0.8 mg/d, vitamin E 7mg/d, selenium yeast 25 μg/d | 1 x 20mg capsule twice daily, NFD. | N/A | 10 days | LPS +/- stimulated peripheral blood cultures:  ? IL-6^2^  ? TNFα^2^  ? Whole blood cell count^2^ |
| Zimecki, 1999 (Poland) [35] | Healthy adults, 25-55 years, **n=27** | bLf capsule, **2mg/d** or **10mg/d** or **50mg d** | 1 x capsule (either 2mg or 10mg or 50mg) daily, NFD | Placebo capsule (lactose, NFD), once daily | 7 days | % Neutrophils in peripheral blood:  PL/2mg/10mg/50mg  ↔/↑^3^/↑^3^/↑^3^  Unstimulated peripheral blood culture:  PL/2mg/10mg/50mg  IL-6 ↔/↔/↓^3^/↔  TNFα ↔/↔/↓^3^/↔ |
| **Author, Year (Country)** | **Participants**, age, n | **Intervention,**  daily dose | **Protocol** | **Control,** daily dose | **Duration** | **Effect of intervention on respiratory tract infections** |
| **ADULTS** | | | | | | |
| Shin, 2018 (Japan) [26] | Healthy adults, 20-65 years, **n=265** | bLf tablet, **60mg/d**, with lactoperoxidase 7.8mg/d and glucose oxidase 7.8mg/d | 1 x 20mg bLf tablet three times daily for sucking during travel or ambulation | N/A | 8 weeks | Influenza infection:  ↔ Incidence  ↔ Duration  Common cold:^5^  ↔ Incidence  ↔ Duration |
| AD, Alzheimer’s disease; bLf, bovine lactoferrin; CD, cluster of differentiation; ID, iron deficient; IDA iron deficiency anemia; IL, interleukin; IPD, individual participant data; Lf, lactoferrin; LPS, lipopolysaccharide; N/A, not applicable; NFD, not further described; NR, not reported; PBMC, peripheral blood mononuclear cell, PHA, phytohemagglutinin; PL, placebo; PMNs, polymorphonuclear leukocytes; T2DM, type 2 diabetes mellitus; TNF, tumor necrosis factor; ↓, significant decrease, ↑, significant increase, ↔, no change. ^1^Significant difference between groups in post-intervention values. ^2^Statistical analysis not performed, individual participant data reported only ^3^Significant change within intervention group compared to baseline values. ^4^Details of cell type and virus stimulation unknown, methods not described. ^5^Common cold symptoms included fever, sore throat, cough, nasal secretion, sniffles, sputum, headache, joint pain, and muscle ache. | | | | | | |

**Supplementary References**

1. Bharadwaj, S, Naidu, TAG, Betageri, GV, Prasadarao, NV, Naidu, AS. Inflammatory responses improve with milk ribonuclease-enriched lactoferrin supplementation in postmenopausal women. Inflamm Res. [Randomized Controlled Trial

Research Support, Non-U.S. Gov't]. 2010 Nov;59(11):971-978.

2. Chen, K, Chai, L, Li, H, Zhang, Y, Xie, HM, Shang, J, Tian, W, Yang, P, Jiang, AC. Effect of bovine lactoferrin from iron-fortified formulas on diarrhea and respiratory tract infections of weaned infants in a randomized controlled trial. Nutrition. 2016 01 Feb;32(2):222-227.

3. Derosa, G, D'Angelo, A, Maffioli, P. Change of some oxidative stress parameters after supplementation with whey protein isolate in patients with type 2 diabetes. Nutrition. 2020 05;73:110700.

4. Dix, C, Wright, O. Bioavailability of a Novel Form of Microencapsulated Bovine Lactoferrin and Its Effect on Inflammatory Markers and the Gut Microbiome: A Pilot Study. Nutrients. 2018 Aug 17;10(8).

5. Fujishima, H, Okada, N, Matsumoto, K, Shimizu, E, Fukuda, S, Tomita, M. Conjunctival injection reduction in patients with atopic keratoconjunctivitis due to synergic effect of bovine enteric-coated lactoferrin in 0.1% tacrolimus ophthalmic suspension. Journal of Clinical Medicine. 2020 October;9(10):1-9.

6. Genazzani, AD, Santagni, S, Ricchieri, F, Campedelli, A, Rattighieri, E, Chierchia, E, Marini, G, Despini, G, Prati, A, Simoncini, T. Myo-inositol modulates insulin and luteinizing hormone secretion in normal weight patients with polycystic ovary syndrome. J Obstet Gynaecol Res. 2014 May;40(5):1353-1360.

7. Ishikado, A, Imanaka, H, Kotani, M, Fujita, A, Mitsuishi, Y, Kanemitsu, T, Tamura, Y, Makino, T. Liposomal lactoferrin induced significant increase of the interferon-alpha (IFN-alpha) producibility in healthy volunteers. Biofactors. [Clinical Trial

Controlled Clinical Trial]. 2004;21(1-4):69-72.

8. Ishikado, A, Uesaki, S, Suido, H, Nomura, Y, Sumikawa, K, Maeda, M, Miyauchi, M, Takata, T, Makino, T. Human trial of liposomal lactoferrin supplementation for periodontal disease. Biological and Pharmaceutical Bulletin. 2010;33(10):1758-1762.

9. Kawakami, H, Park, H, Park, S, Kuwata, H, Shephard, RJ, Aoyagi, Y. Effects of enteric-coated lactoferrin supplementation on the immune function of elderly individuals: A randomised, double-blind, placebo-controlled trial. International Dairy Journal. 2015;47:79-85.

10. King, JC, Jr., Cummings, GE, Guo, N, Trivedi, L, Readmond, BX, Keane, V, Feigelman, S, de Waard, R. A double-blind, placebo-controlled, pilot study of bovine lactoferrin supplementation in bottle-fed infants. J Pediatr Gastroenterol Nutr. 2007 Feb;44(2):245-251.

11. Kozu, T, Iinuma, G, Ohashi, Y, Saito, Y, Akasu, T, Saito, D, Alexander, DB, Iigo, M, Kakizoe, T, Tsuda, H. Effect of orally administered bovine lactoferrin on the growth of adenomatous colorectal polyps in a randomized, placebo-controlled clinical trial. Cancer Prevention Research. 2009;2(11):975-983.

12. Lepanto, MS, Rosa, L, Cutone, A, Conte, MP, Paesano, R, Valenti, P. Efficacy of Lactoferrin Oral Administration in the Treatment of Anemia and Anemia of Inflammation in Pregnant and Non-pregnant Women: An Interventional Study. Frontiers in Immunology. [Clinical Trial

Research Support, Non-U.S. Gov't]. 2018;9:2123.

13. Li, F, Wu, SS, Berseth, CL, Harris, CL, Richards, JD, Wampler, JL, Zhuang, W, Cleghorn, G, Rudolph, CD, Liu, B, Shaddy, DJ, Colombo, J. Improved Neurodevelopmental Outcomes Associated with Bovine Milk Fat Globule Membrane and Lactoferrin in Infant Formula: A Randomized, Controlled Trial. Journal of Pediatrics. 2019 December;215:24-31.e28.

14. Mohamed, WA, Schaalan, MF. Antidiabetic efficacy of lactoferrin in type 2 diabetic pediatrics; controlling impact on PPAR-gamma, SIRT-1, and TLR4 downstream signaling pathway. Diabetol Metab Syndr. 2018;10:89.

15. Mohamed, WA, Salama, RM, Schaalan, MF. A pilot study on the effect of lactoferrin on Alzheimer's disease pathological sequelae: Impact of the p-Akt/PTEN pathway. Biomed Pharmacother. [Randomized Controlled Trial]. 2019 Mar;111:714-723.

16. Motoki, N, Mizuki, M, Tsukahara, T, Miyakawa, M, Kubo, S, Oda, H, Tanaka, M, Yamauchi, K, Abe, F, Nomiyama, T. Effects of Lactoferrin-Fortified Formula on Acute Gastrointestinal Symptoms in Children Aged 12-32 Months: A Randomized, Double-Blind, Placebo-Controlled Trial. Frontiers in pediatrics. 2020;8:233-233.

17. Mulder, AM, Connellan, PA, Oliver, CJ, Morris, CA, Stevenson, LM. Bovine lactoferrin supplementation supports immune and antioxidant status in healthy human males. Nutr Res. 2008 Sep;28(9):583-589.

18. Oda, H, Wakabayashi, H, Tanaka, M, Yamauchi, K, Sugita, C, Yoshida, H, Abe, F, Sonoda, T, Kurokawa, M. Effects of lactoferrin on infectious diseases in Japanese summer: A randomized, double-blinded, placebo-controlled trial. J Microbiol Immunol Infect. 2020 Feb 26.

19. Paesano, R, Pietropaoli, M, Gessani, S, Valenti, P. The influence of lactoferrin, orally administered, on systemic iron homeostasis in pregnant women suffering of iron deficiency and iron deficiency anaemia. Biochimie. 2009 Jan;91(1):44-51.

20. Paesano, R, Berlutti, F, Pietropaoli, M, Pantanella, F, Pacifici, E, Goolsbee, W, Valenti, P. Lactoferrin efficacy versus ferrous sulfate in curing iron deficiency and iron deficiency anemia in pregnant women. BioMetals. [Comparative Study

Research Support, Non-U.S. Gov't

Review]. 2010 Jun;23(3):411-417.

21. Paesano, R, Pietropaoli, M, Berlutti, F, Valenti, P. Bovine lactoferrin in preventing preterm delivery associated with sterile inflammation. Biochemistry & Cell Biology. [Clinical Trial

Research Support, Non-U.S. Gov't]. 2012 Jun;90(3):468-475.

22. Paesano, R, Pacifici, E, Benedetti, S, Berlutti, F, Frioni, A, Polimeni, A, Valenti, P. Safety and efficacy of lactoferrin versus ferrous sulphate in curing iron deficiency and iron deficiency anaemia in hereditary thrombophilia pregnant women: an interventional study. BioMetals. [In Press]. 2014:1-8.

23. Pregliasco, F, Anselmi, G, Fonte, L, Giussani, F, Schieppati, S, Soletti, L. A new chance of preventing winter diseases by the administration of synbiotic formulations. J Clin Gastroenterol. [Randomized Controlled Trial]. 2008 Sep;42 Suppl 3 Pt 2:S224-233.

24. Rosa, L, Lepanto, MS, Cutone, A, Siciliano, RA, Paesano, R, Costi, R, Musci, G, Valenti, P. Influence of oral administration mode on the efficacy of commercial bovine Lactoferrin against iron and inflammatory homeostasis disorders. Biometals. 2020 Jun;33(2-3):159-168.

25. Saraceno, R, Gramiccia, T, Chimenti, S, Valenti, P, Pietropaoli, M, Bianchi, L. Topical lactoferrin can improve stable psoriatic plaque. G Ital Dermatol Venereol. 2014 Jun;149(3):335-340.

26. Shin, K, Wakabayashi, H, Sugita, C, Yoshida, H, Sato, K, Sonoda, T, Yamauchi, K, Abe, F, Kurokawa, M. Effects of orally administered lactoferrin and lactoperoxidase on symptoms of the common cold. Int J Health Sci (Qassim). 2018 Sep-Oct;12(5):44-50.

27. Takeuchi, Y, Yamamura, T, Takahashi, S, Katayose, K, Kohga, S, Takase, M, Imawari, M. Long-term enteral immunonutrition containing lactoferrin in tube-fed bedridden patients: Immunological and nutritional status. Journal of the American College of Nutrition. 2012;31(3):206-213.

28. Tong, PL, West, NP, Cox, AJ, Gebski, VJ, Watts, AM, Dodds, A, de St Groth, BF, Cripps, AW, Shumack, S. Oral supplementation with bovine whey-derived Ig-rich fraction and lactoferrin improves SCORAD and DLQI in atopic dermatitis. Journal of Dermatological Science. [Letter]. 2017 01 Feb;85(2):143-146.

29. Van Splunter, M, Perdijk, O, Fick-Brinkhof, H, Feitsma, AL, Floris-Vollenbroek, EG, Meijer, B, Brugman, S, Savelkoul, HFJ, Van Hoffen, E, Van Neerven, RJJ. Bovine lactoferrin enhances TLR7-mediated responses in plasmacytoid dendritic cells in elderly women: Results from a nutritional intervention study with bovine lactoferrin, GOS and Vitamin D. Frontiers in Immunology. 2018;9(NOV).

30. Vitetta, L, Coulson, S, Beck, SL, Gramotnev, H, Du, S, Lewis, S. The clinical efficacy of a bovine lactoferrin/whey protein Ig-rich fraction (Lf/IgF) for the common cold: a double blind randomized study. Complement Ther Med. 2013 Jun;21(3):164-171.

31. West, NP, Pyne, DB, Cripps, AW, Christophersen, CT, Conlon, MA, Fricker, PA. Gut balance, a synbiotic supplement, increases fecal Lactobacillus paracasei but has little effect on immunity in healthy physically active individuals. Gut Microbes. 2012 May/June;3(3):221-227.

32. Yamauchi, K, Wakabayashi, H, Hashimoto, S, Teraguchi, S, Hayasawa, H, Tomita, M. Effects of orally administered bovine lactoferrin on the immune system of healthy volunteers. Adv Exp Med Biol. 1998;443:261-265.

33. Yen, MH, Chiu, CH, Huang, YC, Lin, TY. Effects of lactoferrin-containing formula in the prevention of enterovirus and rotavirus infection and impact on serum cytokine levels: A randomized trial. Chang Gung Medical Journal. 2011;34(4):395-402.

34. Zimecki, M, Wlaszczyk, A, Cheneau, P, Brunel, AS, Mazurier, J, Spik, G, Kubler, A. Immunoregulatory effects of a nutritional preparation containing bovine lactoferrin taken orally by healthy individuals. Archivum Immunologiae et Therapiae Experimentalis. 1998;46(4):231-240.

35. Zimecki, M, Spiegel, K, Wlaszczyk, A, Kubler, A, Kruzel, ML. Lactoferrin increases the output of neutrophil precursors and attenuates the spontaneous production of TNF-alpha and IL-6 by peripheral blood cells. Archivum Immunologiae et Therapiae Experimentalis. 1999;47(2):113-118.

36. Academy of Nutrition and Dietetics. Evidence Analysis Manual: Steps in the Academy Evidence Analysis Process. 4th ed. Chicago, IL: Academy of Nutrition and Dietetics,; 2016.

37. NHMRC additional levels of evidence and grades for recommendations for developers of guidelines. Canberra (Australia): National Health and Medical Research Council; 2009. Available from: <https://www.nhmrc.gov.au/_files_nhmrc/file/guidelines/developers/nhmrc_levels_grades_evidence_120423.pdf>.
